# Investing in late-stage clinical trials and manufacturing of product candidates for five major infectious diseases: a modelling study of the benefits and costs of investment in three middle-income countries

**DOI:** 10.1016/S2214-109X(22)00206-6

**Published:** 2022-06-14

**Authors:** Marco Schäferhoff, Armand Zimmerman, Mohamed M Diab, Wenhui Mao, Vipul Chowdhary, Davinder Gill, Robert Karanja, Mziwandile Madikizela, Osondu Ogbuoji, Gavin Yamey

**Affiliations:** a Open Consultants, Berlin, Germany; b Center for Policy Impact in Global Health, Duke Global Health Institute, Duke University, Durham, NC, USA; c Policy Cures Research, Sydney, NSW, Australia; d Cambridge, MA, USA; e Villgro Africa, Nairobi, Kenya; f Graduate School of Technology Management, University of Pretoria, South Africa

## Abstract

**Background:**

Investing in late-stage clinical trials, trial sites, and production capacity for new health products could improve access to vaccines, therapeutics, and infectious disease diagnostics in middle-income countries. This study assesses the case for such investment in three of these countries: India, Kenya, and South Africa.

**Methods:**

We applied investment case modelling and assessed how many cases, deaths, and disability-adjusted life years (DALYs) could be averted from the development and manufacturing of new technologies (therapeutics and vaccines) in these countries from 2021 to 2036, for five diseases—HIV, tuberculosis, malaria, pneumonia, and diarrhoeal diseases. We also estimated the economic benefits that might accrue from making these investments and we developed benefit–cost ratios for each of the three middle-income countries. Our modelling applies two investment case perspectives: a societal perspective with all costs and benefits measured at the societal level, and a country perspective to estimate how much health and economic benefit accrues to each middle-income country for every dollar invested in clinical trials and manufacturing by the middle-income country government. For each perspective, we modelled two scenarios: one that considers only domestic health and economic benefits; and one that includes regional health and economic benefits. In the regional scenarios, we assumed that new products developed and manufactured in India would benefit eight countries in south Asia, whereas new products developed and manufactured in Kenya would benefit all 21 countries in the Common Market for Eastern and Southern Africa (COMESA). We also assumed that all 16 countries in the Southern African Development Community (SADC) would benefit from products developed and manufactured in South Africa.

**Findings:**

From 2021 to 2036, product development and manufacturing in Kenya could avert 4·44 million deaths and 206·27 million DALYs in the COMESA region. In South Africa, it could prevent 5·19 million deaths and 253·83 million DALYs in the SADC region. In India, it could avert 9·76 million deaths and 374·42 million DALYs in south Asia. Economic returns would be especially high if new tools were produced for regional markets rather than for domestic markets only. Under a societal perspective, regional returns outweigh investments by a factor of 20·51 in Kenya, 33·27 in South Africa, and 66·56 in India. Under a country perspective, the regional benefit–cost ratios amount to 60·71 in India, 8·78 in Kenya, and 11·88 in South Africa.

**Interpretation:**

Our study supports the creation of regional hubs for clinical trials and product manufacturing compared with narrow national efforts.

**Funding:**

Bill & Melinda Gates Foundation.

## Introduction

Investing in late-stage clinical trials, trial sites, and production capacity for new health products could improve access to vaccines, therapeutics, and diagnostics in middle-income countries. Strengthening trial sites and manufacturing will also contribute to pandemic preparedness and strengthening the response to future outbreaks. The COVID-19 pandemic has shown that low-income and middle-income countries have had to rely mostly on donations of COVID-19 vaccines from high-income countries. As a result, there are now multiple efforts to boost vaccine manufacturing in Africa. WHO, for example, is supporting the creation of African COVID mRNA vaccine technology transfer hubs to scale up production and access to COVID vaccines, with South Africa becoming the first hub. Algeria, Egypt, Morocco, Rwanda, and Senegal have also signed agreements for COVID-19 manufacturing or started production.^1^

In this study, we assessed the case for investing in clinical trials and manufacturing capacity for three middle-income countries: India, Kenya, and South Africa. We modelled the health and economic benefits that would accrue from investments in late-stage clinical trials capacity, trial regulatory systems, and local manufacturing capacity for these countries. We focused on late-stage clinical trials (phase 3 trials) for several reasons. Such trials account for a large share of the overall product development costs and there is a particularly large funding gap for these trials worldwide. Funding for earlier development also remains insufficient, but there have been improvements in financing early-stage development in the past 15 years.^2^, ^3^, ^4^

**Research in context**

**Evidence before this study**

We searched MEDLINE and grey literature (published between Dec 1, 2020, and June 1, 2021) for economic evaluations related to investments in low-income and middle-income countries (LMICs)-based clinical trials and manufacturing of vaccine and therapeutic products targeting infectious diseases. No language restrictions were applied. Key search terms included “HIV”, “tuberculosis”, “malaria”, “pneumonia”, “diarrhea”, “vaccine”, “drug”, “therapeutic”, “diagnostic”, “clinical trials”, “manufacturing”, “cost-effectiveness”, “cost-benefit”, and “LMIC”. Previous studies related to clinical trials and manufacturing of vaccines, drugs, and diagnostics for infectious diseases have focused primarily on the costs of, and current funding for, product research and development. One study calculated that it would cost US$21·0 billion to move 754 infectious disease product candidates in the 2019 research and development pipeline through phase 3 clinical trials. Another study showed that late-stage clinical trials comprise 70% of total vaccine development costs. The 2019 G-FINDER report, the most comprehensive survey of funding for neglected tropical disease research and development, found that 2019 funding for neglected tropical disease basic research and product development amounted to US$3·9 billion. Another study estimated that each US$1·0 invested in phase 3 clinical trials for a selection of infectious diseases could generate US$2·7–5·7 in returns. No studies were found on the health and economic benefits and costs of increasing clinical trial and manufacturing capacity in LMICs.

**Added value of this study**

To our knowledge, this is the first study to estimate the benefit– cost ratios of investments in late-stage clinical trials, manufacturing, and regulation of product candidates for high-burden infectious diseases that disproportionately affect populations from middle-income countries (India, Kenya, and South Africa). Our study uniquely describes the health and economic benefits and costs of investing in clinical trials, manufacturing, and health product regulation from a middle-income country perspective. We show that India, Kenya, and South Africa can generate a substantial return on investment while averting millions of deaths and DALYs. We conclude that large economic and health benefits can be realised if middle-income country governments and donors make coordinated investments in clinical trial and manufacturing capacity.

**Implications of all the available evidence**

Given the novelty of our study, we believe that the results could help inform future investments. The COVID-19 pandemic has shown the need for investments in clinical research and production capacity in middle-income countries. There is more attention being paid towards the development and production of vaccines, especially in developing countries. Many of these countries have been vocal in saying that they urgently need to set up their own manufacturing capacity. Our work, in combination with past research, shows that investing in research and development and local manufacturing should be an immediate priority for countries, from a public health and an economic perspective.

We estimated how many cases, deaths, and disability-adjusted life years (DALYs) could be averted from the development and manufacturing of new therapeutics and vaccines for five infectious diseases: HIV, tuberculosis, malaria, pneumonia, and diarrhoeal diseases. We also estimated the economic benefits that might accrue from making these investments and we develop benefit–cost ratios for each of the three middle-income countries.

## Methods

### Country and disease selection

In selecting middle-income countries, we aimed to include countries that are of different income levels within the middle-income category. For the current 2022 fiscal year, the World Bank defines lower-middle-income countries as those with a gross national income (GNI) per capita between US$1046 and $4095 and upper-middle-income countries as those with a GNI per capita between $4096 and $12 695.^5^ India and Kenya are lower-middle-income countries, and South Africa is an upper-middle-income country. In addition, we selected countries that have a range of capacities in conducting clinical trials and in production of new health products for infectious disease control. In Kenya, product development partnerships and other developers have increasingly funded clinical trials in recent years but there remains a significant potential for expansion.^6^ Kenya has local production for therapeutics but no human vaccine manufacturing capacity yet. Compared with Kenya, South Africa's trial and manufacturing structures are more advanced, including limited capacity for vaccine filling, finishing, and packaging.^7^ India has the strongest trial system of the three middle-income countries chosen and is also a major producer of generic medicines.

The five diseases included in our modelling—HIV, tuberculosis, malaria, pneumonia, and diarrhoeal diseases—collectively account for 12·6% of all 2019 DALYs in India, 35·0% in Kenya, and 38·8% in South Africa (appendix p 1).

This study was approved by Duke University's institutional review board.

### Investment case perspectives

We adopted two perspectives for the investment case modelling: (1) a societal perspective with all costs and benefits measured at the societal level, and (2) a country perspective to estimate how much health and economic benefit accrues to each middle-income country for every dollar invested in clinical trial and manufacturing capacity by the middle-income country government (table 1).

**Table 1:** Investment case perspectives

	**Costs**	**Health benefits**	**Economic benefits**
Societal perspective with regional benefits	Clinical trial capacity building; phase 3 trial costs; NRA costs; and costs for building manufacturing capacity	Regional benefits: DALYs averted, deaths averted, and cases averted	Regional product sales; trial site user fees; regional intellectual property or technology transfer royalties; and regional net treatment costs averted
Societal perspective with domestic benefits	Clinical trial capacity building; phase 3 trial costs; NRA costs; and costs for building manufacturing capacity	Domestic benefits: DALYs averted; deaths averted; and cases averted	Domestic product sales; trial site user fees; domestic intellectual property or technology transfer royalties; and domestic net treatment costs averted
Country perspective with regional benefits	Clinical trial capacity building; phase 3 trial costs (10% of total costs);* NRA costs; costs for building manufacturing capacity (10% of total costs)*	Regional benefits: DALYs averted; deaths averted; and cases averted	Regional product sales (50% of total);† trial site user fees; regional intellectual property or technology transfer royalties (50% of total);† and domestic net treatment costs averted (proportion received as benefit is based on GGHE-D as a percentage of CHE)
Country perspective with domestic benefits	Clinical trial capacity building; phase 3 trial costs (10% of total costs); NRA costs; costs for building manufacturing capacity (10% of total costs)*	Domestic benefits: DALYs averted; deaths averted; cases averted	Domestic product sales (50% of total);† trial site user fees; domestic intellectual property or technology transfer royalties (50% of total);† domestic net treatment costs averted (proportion received as benefit is based on domestic general government health expenditure as a percentage of current health expenditure)

DALYs=disability-adjusted life years. NRA=national regulatory authorities.

* We assumed that in addition to providing 100% of the costs of building clinical trial capacity, each country will contribute 10% of the cost of conducting phase 3 clinical trials and 10% of the cost of building manufacturing capacity; the 90% outstanding costs of conducting clinical trials and the 90% outstanding costs of building manufacturing capacity will be provided by interested investors.

† We assumed an equal partnership arrangement where countries get 50% of any profits arising from sales, intellectual property transfers, or technology transfers of successful products.

The societal perspective seeks to answer the question: how much would society benefit for each US dollar invested in late-stage (phase 3) clinical trials and local manufacturing? This perspective covers all cost components included in our modelling: phase 3 trial costs, the costs for strengthening clinical trial sites, the strengthening of national regulatory authorities (NRAs) as a precondition to develop and manufacture safe and quality-assured medicines and vaccines, and the costs for establishing manufacturing capacity. The societal perspective covers three health benefits—the number of cases, deaths, and DALYs averted—and four economic benefits—the sale of new products, fees charged for the use of trial sites, royalties from intellectual property and technology transfer, and averted treatment costs. Inherently, the societal perspective assumes that a larger group of investors (ie, beyond an individual country) covers all incurred costs.

The country perspective seeks to answer the question: how much would a middle-income country benefit for each dollar the middle-income country government puts into late-stage trials and its own clinical trial and manufacturing structures? The country perspective assumes that the three middle-income countries would invest in their own trial capacity, including their NRAs. Most costs for the late-stage clinical trials themselves, however, are assumed to be covered by external actors, for example by donors, companies, and global funding mechanisms, such as a late-stage clinical trial aggregator. We assumed that the middle-income countries governments would cover 10% of the total late-stage clinical trial costs and 10% of the total investment in manufacturing capacity. The country perspective includes the same three health benefits as the societal perspective (averted number of cases, deaths, and DALYs). Middle-income countries governments might also benefit from fees charged for the use of trial sites. In addition, we assume that the middle-income countries receive 50% of total profits from product sales and tech transfer royalties in our model. This assumption reflects the fact that the country is a co-investor in, and thus does not pay the entirety of, clinical trial and manufacturing costs and, therefore, would not be the sole entity receiving profits from product sales. We also assumed that middle-income country governments receive a proportion of treatment costs averted as a benefit. To estimate the proportion of treatment costs averted that each government receives as a benefit, we use domestic general government health expenditure (GGHE-D) as a percentage of total current health expenditure (CHE) as a proxy (30% for India, 43% for Kenya, and 57% for South Africa).^8^

We considered that the benefits from investing in clinical trials and local production can either be based on domestic returns only or based on the assumption that new health tools will eventually benefit a larger group of countries. Thus, for each perspective, we modelled two scenarios. A first scenario only considers domestic health and economic benefits, whereas a second scenario includes regional health and economic benefits. In the regional scenarios, we assumed that new products developed and manufactured in India would benefit eight countries in south Asia (as defined by the World Bank; ie, Afghanistan, Bangladesh, Bhutan, India, Maldives, Nepal, Pakistan, and Sri Lanka), whereas new products developed and manufactured in Kenya would benefit all 21 countries in the Common Market for Eastern and Southern Africa (COMESA). We also assumed that all 16 countries in the Southern African Development Community (SADC) would benefit from products developed and manufactured in South Africa. Our categorisations exemplify the potential size of regional benefits, but actual benefits might arise from a different group of countries.

### Model specifications

We modelled the costs and the health and economic benefits over a 16-year period between 2021 and 2036. Our model is based on a range of data, which we collected for the purposes of the study. It is also based on assumptions that we made based on an assessment of existing studies and data (appendix p 2).

We did a literature review to collect data on the costs to strengthen clinical trial infrastructure, NRAs, and manufacturing capacity (appendix pp 3–34). First, we assumed that a one-time cost will be incurred to build and equip trial sites (equation 14 in appendix p 37). We also included recurring annual costs for trial site maintenance and clinical research training (equations 15 and 17 in appendix pp 37–38). Second, we used a proxy suggested by the literature to estimate the current quality of NRAs.^9^ The proxy measures the ratio of total pharmaceutical regulation spending to the total pharmaceutical market size. To reach the desired level, the three middle-income countries need to invest in their NRAs until the ratio is equal to the average ratio among the countries with the top ten largest pharmaceutical markets (equation 21 in appendix p 38). Finally, studies show different price tags for building manufacturing capacity.^10^, ^11^, ^12^ We included a total of $250 million to strengthen production capacity in each of the three middle-income countries, assuming that this amount would be sufficient to establish six manufacturing sites, three each for vaccines and therapeutics, which can collectively produce 90 million vaccine doses and 90 million drug doses per year.

We used data from the Portfolio-to-Impact (P2I) tool, a modelling tool that allows users to estimate the costs and probabilities of success for the development of new health technologies.^13^, ^14^ We used the costs per research and development phase, the average length of phase 3 trials, and the probabilities of success from the P2I tool. The tool indicates that two phase 3 trials are sufficient to yield one new product (transition probability of >50%; appendix p 40). Furthermore, we assumed that one new drug and one new vaccine would be developed for HIV, tuberculosis, malaria, and pneumonia, and a new drug for diarrhoea, on the basis of the investments in research and development and manufacturing described. We did not assume that a vaccine for diarrhoea would be developed because there are many different causes for diarrhoeal diseases (various viruses and bacteria) and because it seems difficult to develop one vaccine protecting against all these causes.

We assumed that a country will be able start one trial per product per year until the total number of trials launched is sufficient to achieve one new product for each disease and product type. Due to data limitations, we assumed that each trial site can only run one clinical trial at a time—this is a conservative assumption because advanced sites might well be able to run multiple trials in parallel.

To estimate the health benefits, we assumed that the introduction of a newly developed vaccine reduces the annual incidence of a disease by 10 percentage points per year, until a maximum reduction of 90% in incidence from the protective effect of the vaccine is reached. Incidence data were obtained from the Institute for Health Metrics and Evaluation global burden of disease database.^15^ We assumed a vaccine efficacy of 65% (eg, on the basis of assessment of studies on COVID-19 vaccines [unpublished data from the Center for Policy Impact in Global Health at the Duke Global Health Institute] and other vaccines for infectious diseases, such as rotavirus vaccine^16^ and tuberculosis vaccine^17^). To calculate the public health impact of new therapeutics, we used a similar approach and determined that upon market entry, the introduction of a new drug increases treatment coverage for a disease by 10 percentage points per year until a 90% coverage is reached. Coverage data came from WHO and other sources (appendix pp 3–34). Our assumptions regarding vaccine and therapeutic scale-up at 10 percentage points per year is based on previous published evidence.^18^ Based on the declines in incidence and the increase in treatment coverage, we calculated the deaths and DALYs averted for each disease. Deaths were calculated using case fatality rates extracted through literature review. DALYs were calculated by estimating years of life lost due to death (YLL) and years of life lost due to disability (YLD) per each case of disease. YLL and YLD values were calculated using disability weights and disease durations extracted through literature review (appendix pp 3–34, equations 1–8 in appendix pp 35–36).

We conducted several analyses to model the economic gains. First, we performed a literature review to estimate the costs of procuring and delivering drugs and vaccines (appendix pp 3–34). Based on these cost data and our own estimate on annual cases prevented, treatment costs averted were calculated as the product of cases averted and treatment cost per case, less the sum of the cost of new cases treated and the cost of vaccine procurement (equations 9–13 in appendix pp 36–37).

Second, we calculated the profits resulting from product sales to the domestic or regional markets. To calculate annual product sales, we multiplied the price of therapeutics and vaccines with the estimated number of people receiving the new drug or vaccine in each year according to our health benefit modelling (equation 19 in appendix p 38). However, the total product sales only reflect profits from the sale of doses manufactured by the newly established production plants (six new plants per country). Vaccine and drug doses that cannot be manufactured in these facilities due to capacity limitations will be manufactured by producers from other countries through technology transfer and intellectual property sharing. Finally, the tech transfer involves additional profit from royalties. According to one study, the average royalty rate paid by the world's 15 leading pharmaceutical companies is 11·7% of product sales, with royalty rates of 2·6–5·0% being most frequent.^19^ As royalties in low-income and middle-income countries are often lower compared with high-income countries, we used a conservative rate and calculated tech transfer benefits as 5·0% of sales from all vaccine and drug doses (equation 20 in appendix p 38).

To estimate the benefit–cost ratios, we calculated the ratio of incurred costs and the sum of the economic benefits, which differed across the two perspectives and four scenarios as described. We applied an annual discount rate of 3% to all costs and economic benefits, which is consistent with the discount rate used in other benefit–cost calculations in public health.^20^, ^21^ All costs and financial benefits were converted to 2021 US$ using the Consumer Price Index.^22^

We conducted six sensitivity analyses to account for uncertainty in our parameter estimates (appendix pp 41–42). First, the standard 3·0% discount rate might be inconsistent with low-income and middle-income economies, so we increased the discount rate to 5·0%. Second, we increased the proportion of phase 3 clinical trial and manufacturing costs covered by countries from 10% to 20%. Third, we increased government coverage of phase 3 trial costs and manufacturing costs from 10% to 50%. Fourth, to account for potentially low estimates of phase 3 trial costs in the P2I model, we increased all phase 3 trial costs by 10%. Fifth, we reduced the P2I phase 3 transition probabilities from average to minimum values to account for potential inefficiencies in clinical trial designs. Lastly, while the major focus of our study was on the direct financial gains that result from investments in clinical trial and manufacturing, we added economic productivity to the societal perspective, to capture the longer-term benefits of these investments.

All analyses were conducted using Microsoft Excel (version 16.61).

### Role of the funding source

The funder of this study had no role at all in study design, data analysis, data interpretation, or writing of the report.

## Results

From the societal perspective, during a 16-year period between 2021 and 2036, the investments needed for clinical trials, trial sites, NRAs, and manufacturing range from $1·5 billion in Kenya and India to $1·7 billion in South Africa (table 2). Phase 3 trial costs are the largest investment area, accounting for $1·2 billion per country. Investment needs for NRA strengthening are most substantial in South Africa ($180·91 million), which invests the least in pharmaceutical regulation if current spending for regulation is compared with the size of the country's pharmaceutical market. Our analysis indicates that India has no further NRA investment needs. In Kenya, NRA costs amount to $13·31 million.

**Table 2:** Cost of investing in clinical and manufacturing capacity and phase 3 trials (2021–36) from a societal perspective

	**India**	**Kenya**	**South Africa**
Trial site start-up costs	15·36	26·66	26·66
Trial site maintenance costs	67·11	28·67	28·67
Training costs	0·41	1·73	2·12
NRA costs	0	13·31	180·91
Manufacturing costs	250·00	250·00	250·00
Clinical trial costs	1200·59	1200·59	1200·59
Total costs	1533·46	1520·95	1688·95

Data are 2021 US$ millions. NRA=national regulatory authorities.

Health and economic benefits resulting from these investments are shown in table 3 and the appendix (pp 43–49). Under the regional scenario, the development and manufacturing of new health tools in India would avert 9·76 million deaths and 374·42 million DALYs in south Asia between 2021 and 2036. Almost 1·4 billion cases of the five diseases included in our model could be prevented. Through tools developed and produced in Kenya, 4·44 million deaths and 206·27 million DALYs could be averted in the COMESA countries, whereas 5·19 million deaths and 253·83 million DALYs would be prevented in the SADC countries through tools developed in South Africa (table 3).

**Table 3:** Benefits of investing in clinical trial and manufacturing capacity from a societal perspective, 2021–36

	**India**	**Kenya**	**South Africa**
**Regional scenario**			
Costs,* 2021 US$ millions	1533·46	1520·95	1688·95
Cases averted,† millions	1375·67	773·81	619·73
Deaths averted, millions	9·76	4·44	5·19
DALYs averted, millions	374·42	206·27	253·83
Economic benefits,‡ 2021 US$ millions	102 066·53	31 189·97	56 194·75
BCR	66·56	20·51	33·27
**Domestic scenario**			
Costs,* 2021 US$ millions	1533·46	1520·95	1688·95
Cases averted,† millions	1161·78	61·42	40·05
Deaths averted, millions	8·62	0·42	1·22
DALYs averted, millions	328·48	19·78	60·77
Economic benefits,‡ 2021 US$ millions	42 665·88	1111·54	4810·10
BCR	27·82	0·73	2·85

BCR=benefit–cost ratio. DALYs=disability-adjusted life years.

* Investments in clinical trial capacity building, national regulatory authorities, manufacturing, and in phase 3 trial costs.

† Includes cases from all five conditions modelled: HIV, tuberculosis, malaria, pneumonia, and diarrhoeal diseases.

‡ Product sales, trial site user fees, technology transfer or intellectual property royalties, and net treatment costs averted.

The economic gains of investing in clinical trials and local production in India amount to $102·1 billion over the 16-year timeframe. The benefit–cost ratio for India is high—each $1 dollar invested would return $66·56. The benefit–cost ratios in Kenya and South Africa are lower than in India but still high: for every $1 dollar invested in trials and manufacturing the return would be $20·51 in Kenya and $33·27 South Africa.

Under the domestic scenario, 8·62 million deaths and 328·48 million DALYs would be prevented (table 3). The benefit–cost ratio for India amounts to 27·82. In South Africa, the benefit–cost ratio under the domestic scenario is 2·85, a significantly smaller economic return compared with the regional scenario. 1·22 million deaths and 60·77 million DALYs would be averted in South Africa (table 3). In Kenya, the benefit–cost ratio is 0·73, a benefit–cost ratio less than 1. The number of averted deaths amount to 0·42 million and DALYs to 19·78 million in Kenya (table 3).

According to our second modelling perspective, the country perspective, over 16 years, investment needs range from $215·42 million in Kenya, to $227·94 million in India, and $383·42 million in South Africa (table 4).

**Table 4:** Costs of investing in clinical trial and manufacturing capacity (2021–36) from a country perspective

	**India**	**Kenya**	**South Africa**
Trial site start-up costs	15·36	26·66	26·66
Trial site maintenance costs	67·11	28·67	28·67
Training costs	0·41	1·73	2·12
NRA costs	0	13·31	180·91
Costs for building manufacturing capacity (10% of total costs)	25·00	25·00	25·00
Clinical trials costs (10% of total costs)	120·06	120·06	120·06
Total costs	227·94	215·42	383·42

Data are 2021 US$ millions. NRA=national regulatory authorities.

Economic and health benefits accruing from the perspective of the governments of the middle-income countries are shown in table 5 (as described in the Methods section, the health benefits do not differ from those of the societal perspective). Under the regional scenario, the benefit–cost ratio for Kenya was 8·78 and 11·88 for South Africa. The benefit–cost ratio in India is 60·71, and is thus also lower compared with the societal perspective.

**Table 5:** Benefits of investing in clinical trial and manufacturing capacity from a country perspective, 2021–36

	**India**	**Kenya**	**South Africa**
**Regional scenario**			
Costs,* 2021 US$ millions	227·94	215·42	383·42
Cases averted,† millions	1375·67	773·81	619·73
Deaths averted, millions	9·76	4·44	5·19
DALYs averted, millions	374·42	206·27	253·83
Economic benefits,‡ 2021 US$ millions	13 837·67	1892·02	4556·25
BCR	60·71	8·78	11·88
**Domestic scenario**			
Costs,* 2021 US$ millions	227·94	215·42	383·42
Cases averted,† millions	1161·78	61·42	40·05
Deaths averted, millions	8·62	0·42	1·22
DALYs averted, millions	328·48	19·78	60·77
Economic benefits,‡ 2021 US$ millions	14 227·46	540·57	2687·60
BCR	62·42	2·51	7·01

BCR=benefit–cost ratio. DALYs=disability-adjusted life years.

* Investments in clinical trial capacity building and national regulatory authorities and 10% of total costs for manufacturing and phase 3 trials.

† Includes cases from all five conditions modelled: HIV, tuberculosis, malaria, pneumonia, and diarrhoeal diseases.

‡ Trial site user fees, 50% of profit resulting from product sales and technology transfer or intellectual property royalties, and net treatment costs averted (domestic general government health expenditure as a percentage of current health expenditure).

The domestic scenario shows that the economic benefits resulting from domestic markets only are comparatively small. In Kenya, each dollar invested by the government would return $2·51, and in South Africa, each dollar would return $7·01. In India, the benefit–cost ratio is 62·42, which is higher than the regional benefit–cost ratio.

The results of the sensitivity analyses are shown in the appendix (pp 41–42).

## Discussion

Our study provides evidence on the health benefits and the economic returns of investing in clinical trials and manufacturing capacity in India, Kenya, and South Africa. It shows that investments in trials and manufacturing would have a substantial public health impact, especially if products developed and manufactured in these three middle-income countries are exported to a larger group of countries. Under the regional scenario, product development and manufacturing in Kenya could avert 4·44 million deaths and 206·27 million DALYs in the COMESA region over the period 2021–36. In South Africa, such investments could prevent 5·19 million deaths and 253.83 million DALYs in the SADC region. In south Asia, 9·76 million deaths and 374·42 million DALYs could be averted through such investments.

We provide a conservative estimate of the health benefits. Our study is focused on the health benefits of new health tools for five diseases. However, investments in trial sites and manufacturing will be useful for a much broader range of infectious and non-communicable diseases and other health challenges, such as antimicrobial resistance. Hence, the benefits resulting from these investments would likely be higher. There are also other benefits that are hard to quantify due to insufficient data. Although there is little evidence, lower prices for medicines due to local production should also help to increase access to medicines, to mitigate health inequalities within middle-income countries, and to make progress towards universal health coverage. Middle-income countries would be enabled to leverage their own research and manufacturing capacity in times of health crisis rather than relying on external support.

Our results also show that investing in clinical trials and local production pays off from an economic perspective. Economic returns would be especially high if new tools were produced for regional markets rather than for domestic markets only: under the societal perspective, regional returns outweigh investments by a factor of 21 in Kenya, and by a factor of 33 in South Africa. In India, the returns are as high as about $67 per dollar invested. The high economic returns in India result from the large population size and high incidence of the five diseases in south Asia, which translate into substantial product sales and tech transfer royalties. The net savings from treatment costs averted are also large. In addition, India recently reformed its NRA and has therefore no further NRA investment needs.^23^ The ecomonic returns in Kenya and South Africa are also high but, because of the comparatively smaller number of annual cases, the benefit–cost ratios for Kenya and South Africa are lower than in India. Under the regional scenario of the societal perspective, the economic retruns are substantial even if compared with other investments in global health. The *Lancet* Commission on Investing in Health, for example, found that each dollar invested in a grand convergence—a reduction in infectious, child, and maternal mortality to rates seen in the best-performing middle-income countries—would return $9–20.^20^

However, the benefit–cost ratios, especially for Kenya and South Africa, change substantially if only domestic markets are targeted. In South Africa, the benefit–cost ratio drops to 2·85 from 33·27, whereas the benefit–cost ratio for Kenya declines to 0·73 from 20·51, if only domestic markets are considered. A benefit–cost ratio smaller than 1 means that the investments do not pay off from an economic perspective (ie, if the substantial public health benefits are dismissed).

This pattern also arises from the country perspective—the investments of middle-income countries governments pay off when regional rather than domestic markets are targeted. Compared with the societal perspective, the benefit–cost ratios are also lower because middle-income governments only accrue a proportion of total averted net treatment costs. Only for India, the returns are similar to the regional south Asian scenario as India accounts for a large share of the regional incidence. In addition, on average, treatment coverage in India is lower than treatment coverage in south Asia. Lower treatment coverage rates in India under the domestic scenario result in larger product sales and, therefore, higher net benefits, increasing the domestic benefit–cost ratio over the regional benefit–cost ratio.

Our study, therefore, supports the creation of regional hubs for clinical trials and product manufacturing compared with narrow national efforts. Creating regional manufacturing hubs is particularly relevant for Africa—currently 99% of Africa's vaccines are imported because of very limited vaccine upstream production.^24^

There are three key limitations to our study. First, as with all models, our modelling was based on several assumptions, such as the potential impact of new health technologies. We based all assumptions on the best available data, but there will always be uncertainties around these assumptions. Second, our study only estimates the benefits of new health products for five major infectious diseases. Investments in trial sites, NRAs, and manufacturing would likely benefit a much larger range of infectious diseases and non-communicable diseases, for which incidences are increasing in all regions because of demographic changes and exposure to risk factors, such as changes in diet and physical activity. Our study might thus underestimate the returns. Finally, our regional categorisations only indicate the potential of the regional health and economic benefits arising from investing in trial sites and local production. India might, for example, sell its products well beyond south Asia.

## Data sharing

Data used in the modelling are available in the appendix, which also includes the data sources. The authors are willing to share more specific data on reasonable request. Please email Marco Schäferhoff (mschaeferhoff@openconsultants.org) and Armand Zimmerman (armand.zimmerman@duke.edu).

## Declaration of interests

All authors report grants from the Bill & Melinda Gates Foundation during the conduct of the study. RK also serves as chairman of the steering committee of the Coalition for Health Research & Development, a policy and advocacy coalition that is funded by the Bill & Melinda Gates Foundation. This is a voluntary position that does not have any direct renumeration.
