# Supplementary material for: Investing in late-stage clinical trials and manufacturing of product candidates for five major infectious diseases: a modelling study of the benefits and costs of investment in three middle-income countries
Source: Lancet Glob Health. 2022 Jun 14;10(7):e1045–52. doi: 10.1016/S2214-109X(22)00206-6 (PMC9210258; doi:10.1016/S2214-109X(22)00206-6)
Supplement: Supplementary appendix [file mmc1.pdf]

THE LANCET
Global Health

Supplementary appendix

This appendix formed part of the original submission and has been peer reviewed. We post it as supplied by the authors.

Supplement to: Schäferhoff M, Zimmerman A, Diab MM, et al. Investing in late-stage clinical trials and manufacturing of product candidates for five major infectious diseases: a modelling study of the benefits and costs of investment in three middle-income countries. *Lancet Glob Health* 2022; **10:** e1045–52.

Annexes

Annex 1: Annual incidence of the five diseases included in the model (2019)

	**Annual 2019 incidence (in million cases)**		
**Disease**	**India**	**Kenya**	**South Africa**
HIV	0.07	0.07	0.37
TB	3	0.11	0.22
Malaria	9.9	5.3	0.14
Pneumonia	150	3	4.8
Diarrheal diseases	1,700	51	49

Annex 2: Model assumptions

Building clinical trial capacity	A one-time cost will be incurred per trial site, to build and equip the trial site (Source: Authors' calculation based on literature review).
Building clinical trial capacity	Once a site is built, a recurring annual cost will be incurred per site to maintain and further develop capacity (Source: Authors' calculation based on literature review).
Building clinical trial capacity	Countries will make a recurring annual investment in clinical infectious disease research training to sustain capacity (Source: Authors' calculation based on literature review).
Strengthening NRAs	Countries will invest in regulation to ensure that its ratio of total pharmaceutical regulation spending to total pharmaceutical market size is equal to the average ratio among the countries with the top 10 largest pharmaceutical markets (Source: Authors' calculation based on literature review).
Clinical trials	Costs for phase III trials for vaccines and drugs included (Source: P2I).
Clinical trials	Phase III trials for at least two candidate products of the same product-archetype are sufficient to yield one product launch. P2I indicates a transition probability >50% for drugs and vaccines (Source: P2I).
Clinical trials	Average drug and vaccine clinical trial duration is three years (Data source: P2I).
Clinical trials	One vaccine and one new drug will be developed for HIV, TB, malaria, pneumonia. For diarrhea, a new drug will be developed.
Clinical trials	A country will start one trial per product per year, until the total number of trials started is enough to guarantee one launch per product. In this case, two years.
Clinical trials	Each trial site can only run one clinical trial at a time.
Clinical trials	No lag between investing in clinical trial capacity and starting a clinical trial.
Manufacturing capacity	Total investment of $250 million investment per country for strengthening manufacturing capacity. Equivalent to building three new plants for vaccines and drugs each (Source: Authors' calculation based on literature review).
Health benefits	New vaccine launch will reduce the annual regional or domestic incidence of a disease by 10 percentage points per year, until a maximum reduction of 90% reduction in incidence from the protective effect of vaccines is reached.
Health benefits	Vaccine efficacy: We assume a successfully developed vaccine will be 65% effective in preventing the disease.
Health benefits	New drug launch will increase regional or domestic treatment coverage for a disease by 10 percentage points per year, until a coverage of 90% is reached.
Economic returns (general)	A product will launch in the year following the year of clinical trial completion (i.e., if clinical trial ends in 2023, product will launch in 2024).
Economic returns (trial site fees)	Countries/investors will receive a fixed annual return per trial site, for each year that a trial site is in use. This return represents the amount paid to the investor by a contract research organization.
Economic returns (product sales)	Each product launch will generate profits in the form of product sales to the domestic or regional market (COMESA; SADC; south Asia). Product sales are only accrued from doses manufactured in the newly created production plants (total volume: 90 million vaccines and drug doses per year).
Economic returns (IP & tech transfer royalties)	Each product launch will result in one biopharmaceutical license that involves a return from tech transfer/IP royalties. Tech transfer royalties are calculated as 5.0% of product sales resulting from tech transfer arrangements with manufacturers from other countries. Benefits from tech transfers were only included when demand for the successful candidate exceeded the newly installed manufacturing capacity of 90 million vaccine doses and 90 million drug doses per year.
Economic returns (net treatment costs averted)*	Treatment costs averted were calculated as the product of cases averted and treatment cost per case, minus the sum of the cost of new cases treated and the cost of vaccine procurement.
Other	Applied discount rate of 3% on costs and economic benefits.
Other	All costs and financial benefits were converted to 2021 US$ using the Consumer Price Index (CPI).

*Only covered under the societal perspective.

Annex 3: Data and data sources

Estimates and Data Sources used for India Investment Case (Currencies Reported in 2021 US$)

Metric	Average Value (Range)	Source
Trial site start-up cost	$891,905.95 (NA)	In-country consultant.
Trial site annual maintenance cost	$308,736.67 (NA)	In-country consultant
Annual investment in training	$32,574.01 (NA)	https://reporter.nih.gov/search/_-SaErbankGWfMJIzrYJlw/project-details/9265341
Vaccine phase 3 clinical trial cost	$122,210,000.00 (111,100,000.00-133,320,000.00)	Terry RF, Yamey G, Miyazaki-Krause R *et al.* Funding global health product R&D: the Portfolio-To-Impact Model (P2I), a new tool for modelling the impact of different research portfolios [version 2; peer review: 2 approved]. *Gates Open Res* 2018, **2**:24 (https://doi.org/10.12688/gatesopenres.12816.2)
Drug phase 3 clinical trial cost	$27,720,000.00 (17,610,000.00-59,530,000.00)	Terry RF, Yamey G, Miyazaki-Krause R *et al.* Funding global health product R&D: the Portfolio-To-Impact Model (P2I), a new tool for modelling the impact of different research portfolios [version 2; peer review: 2 approved]. *Gates Open Res* 2018, **2**:24 (https://doi.org/10.12688/gatesopenres.12816.2)
Trial site annual user fee	$1,118,851.30 (NA)	https://aspe.hhs.gov/report/examination-clinical-trial-costs-and-barriers-drug-development

Technology transfer royalties as a percentage of product sales	11.69% (1.25-48.80)	Borshell, N., Dawkes, A. Pharmaceutical royalties in licensing deals: No place for the 25 per cent rule of thumb. *J Commer Biotechnol* **16,** 8–16 (2010). https://doi.org/10.1057/jcb.2009.13
Product sale profits as a percentage of treatment costs	20.00% (NA)	https://www.mckinsey.com/industries/public-and-social-sector/our-insights/should-sub-saharan-africa-make-its-own-drugs
HIV disability weight	0.08 (0.01-0.58)	Global Burden of Disease Collaborative Network. Global Burden of Disease Study 2019 (GBD 2019) Disability Weights. Seattle, United States of America: Institute for Health Metrics and Evaluation (IHME), 2020.
TB disability weight	0.33 (0.33-0.33)	Global Burden of Disease Collaborative Network. Global Burden of Disease Study 2019 (GBD 2019) Disability Weights. Seattle, United States of America: Institute for Health Metrics and Evaluation (IHME), 2020.
Malaria disability weight	0.13 (0.01-0.40)	Global Burden of Disease Collaborative Network. Global Burden of Disease Study 2019 (GBD 2019) Disability Weights. Seattle, United States of America: Institute for Health Metrics and Evaluation (IHME), 2020.
Pneumonia disability weight	0.13 (0.13-0.13)	Global Burden of Disease Collaborative Network. Global Burden of Disease Study 2019 (GBD 2019) Disability Weights. Seattle, United States of America: Institute for Health Metrics and Evaluation (IHME), 2020.

Diarrheal disease disability weight	0.17 (0.07-0.25)	Global Burden of Disease Collaborative Network. Global Burden of Disease Study 2019 (GBD 2019) Disability Weights. Seattle, United States of America: Institute for Health Metrics and Evaluation (IHME), 2020.
HIV incidence	73,445.33 (44,494.79-115,564.79)	Institute for Health Metrics and Evaluation (IHME). GBD Compare. Seattle, WA: IHME, University of Washington, 2015. Available from http://vizhub.healthdata.org/gbd-compare. (Accessed [June 24, 2021])
HIV incidence (South Asia region)	87,909.61 (50,076.57-165,037.66)	Institute for Health Metrics and Evaluation (IHME). GBD Compare. Seattle, WA: IHME, University of Washington, 2015. Available from http://vizhub.healthdata.org/gbd-compare. (Accessed [June 24, 2021])
HIV treatment cost per patient per year	$632.62 (427.05-838.19)	Sharma A, Prinja S, Sharma A, Gupta A, Arora SK. Cost of antiretroviral treatment for HIV patients in two centres of North India. International Journal of STD & AIDS. 2019;30(8):769-778. doi:10.1177/0956462419839852
HIV drug cost per patient per year	$432.16 (277.58-586.73)	Sharma A, Prinja S, Sharma A, Gupta A, Arora SK. Cost of antiretroviral treatment for HIV patients in two centres of North India. International Journal of STD & AIDS. 2019;30(8):769-778. doi:10.1177/0956462419839852

HIV treatment coverage	56.00% (NA)	https://www.avert.org/professionals/hiv-around-world/asia-pacific/india
HIV treatment coverage (South Asia region)	63.00% (NA)	https://www.avert.org/professionals/hiv-around-world/asia-pacific/overview
TB incidence	3,043,255.80 (2,589,993.47-3,568,849.22)	Institute for Health Metrics and Evaluation (IHME). GBD Compare. Seattle, WA: IHME, University of Washington, 2015. Available from http://vizhub.healthdata.org/gbd-compare. (Accessed [June 24, 2021])
TB incidence (Africa region)	3,814,554.04 (3,271,156.40-4,428,901.37)	Institute for Health Metrics and Evaluation (IHME). GBD Compare. Seattle, WA: IHME, University of Washington, 2015. Available from http://vizhub.healthdata.org/gbd-compare. (Accessed [June 24, 2021])
TB treatment cost per patient	$1,114.98 (35.45-3,578.17)	Floyd, K., Arora, V. K., Murthy, K. J., Lonnroth, K., Singla, N., Akbar, Y., Zignol, M., & Uplekar, M. (2006). Cost and cost-effectiveness of PPM-DOTS for tuberculosis control: evidence from India. *Bulletin of the World Health Organization*, *84*(6), 437–445. https://doi.org/10.2471/blt.05.024109

		John D, Chatterjee P, Murthy S, Bhat R, Musa BM. Cost effectiveness of decentralised care model for managing MDR-TB in India. Indian J Tuberc. 2018 Jul;65(3):208-217. doi: 10.1016/j.ijtb.2017.08.031. Epub 2017 Sep 28. PMID: 29933862.
TB drug cost per patient	$54.54 (13.29-97.16)	Floyd, K., Arora, V. K., Murthy, K. J., Lonnroth, K., Singla, N., Akbar, Y., Zignol, M., & Uplekar, M. (2006). Cost and cost-effectiveness of PPM-DOTS for tuberculosis control: evidence from India. *Bulletin of the World Health Organization*, *84*(6), 437–445. https://doi.org/10.2471/blt.05.024109<br><br>Deo, S., Jindal, P., Gupta, D., Khaparde, S., Rade, K., Sachdeva, K. S., Vadera, B., Shah, D., Patel, K., Dave, P., Chopra, R., Jha, N., Papineni, S., Vijayan, S., & Dewan, P. (2019). What would it cost to scale-up private sector engagement efforts for tuberculosis care? Evidence from three pilot programs in India. *PloS one*, *14*(6), e0214928. https://doi.org/10.1371/journal.pone.0214928
TB treatment coverage	58.00% (NA)	https://www.who.int/data/gho/data/indicators/indicator-details/GHO/tuberculosis-effective-treatment-coverage-(-)
TB treatment coverage (South Asia region)	63.00% (NA)	https://www.who.int/data/gho/data/indicators/indicator-details/GHO/tuberculosis-effective-treatment-coverage-(-)

Malaria incidence	9,894,049.83 (4,942,728.77-18,709,026.18)	Institute for Health Metrics and Evaluation (IHME). GBD Compare. Seattle, WA: IHME, University of Washington, 2015. Available from http://vizhub.healthdata.org/gbd-compare. (Accessed [June 24, 2021])
Malaria incidence (South Asia region)	12,234,653.07 (6,579,005.21-22,083,792.09)	Institute for Health Metrics and Evaluation (IHME). GBD Compare. Seattle, WA: IHME, University of Washington, 2015. Available from http://vizhub.healthdata.org/gbd-compare. (Accessed [June 24, 2021])
Malaria treatment cost per patient	$35.79 (34.12-38.55)	Gogtay, N. J., Kadam, V. S., Desai, S., Kamtekar, K. D., Dalvi, S. S., & Kshirsagar, N. A. (2003). A cost-effectiveness analysis of three antimalarial treatments for acute, uncomplicated Plasmodium falciparum malaria in Mumbai, India. *The Journal of the Association of Physicians of India*, *51*, 877–879.
Malaria drug cost per patient	$2.24 (0.05-7.20)	Kumar, L., Dinkar, J.K., Mohan, L., and Dikshit, H. Cost variation of antimalarial drugs available in India. (2017). International Journal of Research in Medical Sciences. doi: http://dx.doi.org/10.18203/2320-6012.ijrms20173981
Malaria treatment coverage	90.00% (NA)	https://www.who.int/malaria/publications/country-profiles/profile_ind_en.pdf?ua=1

Malaria treatment coverage (South Asia region)	91.00% (NA)	https://www.who.int/malaria/publications/country-profiles/profile_ind_en.pdf?ua=1<br><br>https://www.who.int/malaria/publications/country-profiles/profile_npl_en.pdf<br><br>https://www.who.int/malaria/publications/country-profiles/profile_lka_en.pdf?ua=1<br><br>https://www.who.int/malaria/publications/country-profiles/profile_pak_en.pdf<br><br>https://www.who.int/malaria/publications/country-profiles/profile_afg_en.pdf<br><br>https://www.who.int/malaria/publications/country-profiles/profile_bgd_en.pdf?ua=1
Pneumonia incidence	1480,347,208.15 (138,305,022.09-159,263,464.09)	Institute for Health Metrics and Evaluation (IHME). GBD Compare. Seattle, WA: IHME, University of Washington, 2015. Available from http://vizhub.healthdata.org/gbd-compare. (Accessed [June 24, 2021])

Pneumonia incidence (South Asia region)	174,927,530.66 (163,067,793.51-187,409,925.13)	Institute for Health Metrics and Evaluation (IHME). GBD Compare. Seattle, WA: IHME, University of Washington, 2015. Available from http://vizhub.healthdata.org/gbd-compare. (Accessed [June 24, 2021])
Pneumonia treatment cost per patient	$201.80 (NA)	Saha, L., Kaur, S., Khosla, P., Kumari, S., & Rani, A. (2017). Pharmacoeconomic Analysis of Drugs Used in the Treatment of Pneumonia in Paediatric Population in a Tertiary Care Hospital in India-A Cost-of-Illness Study. *Medical sciences (Basel, Switzerland)*, *5*(4), 33. https://doi.org/10.3390/medsci5040033
Pneumonia drug cost per patient	$63.02 (NA)	Saha, L., Kaur, S., Khosla, P., Kumari, S., & Rani, A. (2017). Pharmacoeconomic Analysis of Drugs Used in the Treatment of Pneumonia in Paediatric Population in a Tertiary Care Hospital in India-A Cost-of-Illness Study. *Medical sciences (Basel, Switzerland)*, *5*(4), 33. https://doi.org/10.3390/medsci5040033
Pneumonia treatment coverage	60.00% (NA)	Knowles, R., Sharland, M., Hsia, Y., Magrini, N., Moja, L., Siyam, A., & Tayler, E. (2020). Measuring antibiotic availability and use in 20 low- and middle-income countries. *Bulletin of the World Health Organization*, *98*(3), 177–187C. https://doi.org/10.2471/BLT.19.241349

Pneumonia treatment coverage (South Asia region)	82.00% (NA)	Knowles, R., Sharland, M., Hsia, Y., Magrini, N., Moja, L., Siyam, A., & Tayler, E. (2020). Measuring antibiotic availability and use in 20 low- and middle-income countries. *Bulletin of the World Health Organization*, *98*(3), 177–187C. https://doi.org/10.2471/BLT.19.241349
Diarrheal disease incidence	1,679,827,027.98 (1,533,079,824.92-1,838,631,091.11)	Institute for Health Metrics and Evaluation (IHME). GBD Compare. Seattle, WA: IHME, University of Washington, 2015. Available from http://vizhub.healthdata.org/gbd-compare. (Accessed [June 24, 2021])
Diarrheal disease incidence (South Asia region)	2,232,103,062.90 (2,033,280,407.15-2,447,991,965.86)	Institute for Health Metrics and Evaluation (IHME). GBD Compare. Seattle, WA: IHME, University of Washington, 2015. Available from http://vizhub.healthdata.org/gbd-compare. (Accessed [June 24, 2021])
Diarrheal disease treatment cost per patient	$8.69 (NA)	Baral, R., Nonvignon, J., Debellut, F. *et al.* Cost of illness for childhood diarrhea in low- and middle-income countries: a systematic review of evidence and modelled estimates. *BMC Public Health* **20,** 619 (2020). https://doi.org/10.1186/s12889-020-08595-8
Diarrheal disease drug cost per patient	$3.90 (NA)	Baral, R., Nonvignon, J., Debellut, F. *et al.* Cost of illness for childhood diarrhea in low- and middle-income countries: a systematic review of evidence and modelled estimates. *BMC*

		Public Health **20,** 619 (2020). https://doi.org/10.1186/s12889-020-08595-8
Diarrheal disease treatment coverage	58.00% (NA)	Local Burden of Disease Diarrhoea Collaborators (2020). Mapping geographical inequalities in oral rehydration therapy coverage in low-income and middle-income countries, 2000-17. *The Lancet. Global health*, *8*(8), e1038–e1060. https://doi.org/10.1016/S2214-109X(20)30230-8
Diarrheal disease treatment coverage (South Asia region)	55.00% (NA)	Local Burden of Disease Diarrhoea Collaborators (2020). Mapping geographical inequalities in oral rehydration therapy coverage in low-income and middle-income countries, 2000-17. *The Lancet. Global health*, *8*(8), e1038–e1060. https://doi.org/10.1016/S2214-109X(20)30230-8
Percentage of all drugs in India that are imported	68.00% (NA)	https://timesofindia.indiatimes.com/india/india-imports-68-of-its-bulk-drugs-from-china/articleshow/76485141.cms
Employment rate	43.00% (NA)	https://ilostat.ilo.org/
Annual minimum wage	$698.09 (NA)	https://ilostat.ilo.org/

Domestic general government health expenditure (GGHE-D) as a percent of current health expenditure (CHE)	30.00% (NA)	https://apps.who.int/nha/database

Estimates and Data Sources used for Kenya Investment Case (Currencies Reported in 2021 US$)

Metric	Average Value (Range)	Source
Trial site start-up cost	$1,548,218.93 (NA)	https://www.idrc.ca/sites/default/files/sp/Documents%20EN/HIV-AIDS-Prevention-Trials-Capacity-Building-Grants-booklet.pdf
Trial site annual maintenance cost	$131,885.32 (103,214.60-137,619.46)	https://www.idrc.ca/sites/default/files/sp/Documents%20EN/HIV-AIDS-Prevention-Trials-Capacity-Building-Grants-booklet.pdf
Annual investment in training	$137,619.46 (NA)	https://www.idrc.ca/sites/default/files/sp/Documents%20EN/HIV-AIDS-Prevention-Trials-Capacity-Building-Grants-booklet.pdf
Vaccine phase 3 clinical trial cost	$122,210,000.00 (111,100,000.00-133,320,000.00)	Terry RF, Yamey G, Miyazaki-Krause R *et al.* Funding global health product R&D: the Portfolio-To-Impact Model (P2I), a new tool for modelling the impact of different research portfolios [version 2; peer review: 2 approved]. *Gates Open Res* 2018, **2**:24 (https://doi.org/10.12688/gatesopenres.12816.2)
Drug phase 3 clinical trial cost	$27,720,000.00 (17,610,000.00-59,530,000.00)	Terry RF, Yamey G, Miyazaki-Krause R *et al.* Funding global health product R&D: the Portfolio-To-Impact Model (P2I), a new tool for modelling the impact of different research portfolios [version 2; peer review: 2 approved]. *Gates Open Res* 2018, **2**:24 (https://doi.org/10.12688/gatesopenres.12816.2)

Trial site annual user fee	$1,118,851.30 (NA)	https://aspe.hhs.gov/report/examination-clinical-trial-costs-and-barriers-drug-development
Technology transfer royalties as a percentage of product sales	11.69% (1.25-48.80)	Borshell, N., Dawkes, A. Pharmaceutical royalties in licensing deals: No place for the 25 per cent rule of thumb. *J Commer Biotechnol* **16,** 8–16 (2010). https://doi.org/10.1057/jcb.2009.13
Product sale profits as a percentage of treatment costs	20.00% (NA)	https://www.mckinsey.com/industries/public-and-social-sector/our-insights/should-sub-saharan-africa-make-its-own-drugs
HIV disability weight	0.08 (0.01-0.58)	Global Burden of Disease Collaborative Network. Global Burden of Disease Study 2019 (GBD 2019) Disability Weights. Seattle, United States of America: Institute for Health Metrics and Evaluation (IHME), 2020.
TB disability weight	0.33 (0.33-0.33)	Global Burden of Disease Collaborative Network. Global Burden of Disease Study 2019 (GBD 2019) Disability Weights. Seattle, United States of America: Institute for Health Metrics and Evaluation (IHME), 2020.
Malaria disability weight	0.13 (0.01-0.40)	Global Burden of Disease Collaborative Network. Global Burden of Disease Study 2019 (GBD 2019) Disability Weights. Seattle, United States of America: Institute for Health Metrics and Evaluation (IHME), 2020.

Pneumonia disability weight	0.13 (0.13-0.13)	Global Burden of Disease Collaborative Network. Global Burden of Disease Study 2019 (GBD 2019) Disability Weights. Seattle, United States of America: Institute for Health Metrics and Evaluation (IHME), 2020.
Diarrheal disease disability weight	0.17 (0.07-0.25)	Global Burden of Disease Collaborative Network. Global Burden of Disease Study 2019 (GBD 2019) Disability Weights. Seattle, United States of America: Institute for Health Metrics and Evaluation (IHME), 2020.
HIV incidence	67,620.99 (50,604.23-88,256.79)	Institute for Health Metrics and Evaluation (IHME). GBD Compare. Seattle, WA: IHME, University of Washington, 2015. Available from http://vizhub.healthdata.org/gbd-compare. (Accessed [June 24, 2021])
HIV incidence (Africa region)	1,306,590.80 (1,093,181.64-1,576,684.33)	Institute for Health Metrics and Evaluation (IHME). GBD Compare. Seattle, WA: IHME, University of Washington, 2015. Available from http://vizhub.healthdata.org/gbd-compare. (Accessed [June 24, 2021])
HIV treatment cost per patient per year	$303.61 (255.51-353.92)	U.S. Centers for Diseases Control and Kenya Ministry of Health, (2013), The Cost of Comprehensive HIV Treatment in Kenya. Report of a Cost Study of HIV Treatment Programs in Kenya. Atlanta, GA (USA) and Nairobi, Kenya.<br><br>Larson, B. A., Bii, M., Henly-Thomas, S., McCoy, K., Sawe, F., Shaffer, D., & Rosen, S. (2013). ART treatment costs and retention

		in care in Kenya: a cohort study in three rural outpatient clinics. *Journal of the International AIDS Society*, *16*(1), 18026. https://doi.org/10.7448/IAS.16.1.18026
HIV drug cost per patient per year	$139.75 (NA)	U.S. Centers for Diseases Control and Kenya Ministry of Health, (2013), The Cost of Comprehensive HIV Treatment in Kenya. Report of a Cost Study of HIV Treatment Programs in Kenya. Atlanta, GA (USA) and Nairobi, Kenya.
HIV treatment coverage	74% (65-86)	https://www.unaids.org/en/regionscountries/countries/kenya
HIV treatment coverage (Africa region)	56.00% (NA)	https://aidsinfo.unaids.org/
TB incidence	113,515.42 (96,815.33-88,256.79)	Institute for Health Metrics and Evaluation (IHME). GBD Compare. Seattle, WA: IHME, University of Washington, 2015. Available from http://vizhub.healthdata.org/gbd-compare. (Accessed [June 24, 2021])
TB incidence (Africa region)	2,198,950.36 (1,921,392.55-2,525,066.04)	Institute for Health Metrics and Evaluation (IHME). GBD Compare. Seattle, WA: IHME, University of Washington, 2015. Available from http://vizhub.healthdata.org/gbd-compare. (Accessed [June 24, 2021])

TB treatment cost per patient	$2,624.65 (212.66-5,036.63)	Vassall, Anna. Post 2015 Development Agenda: Kenya Perspectives, Tuberculosis. Copenhagen Consensus.<br><br>https://www.copenhagenconsensus.com/publication/post-2015-consensus-health-perspective-tuberculosis-vassall
TB drug cost per patient	$212.66 (NA)	Vassall, Anna. Post 2015 Development Agenda: Kenya Perspectives, Tuberculosis. Copenhagen Consensus.<br><br>https://www.copenhagenconsensus.com/publication/post-2015-consensus-health-perspective-tuberculosis-vassall
TB treatment coverage	52.00% (NA)	https://www.who.int/data/gho/data/indicators/indicator-details/GHO/tuberculosis-effective-treatment-coverage-(-)
TB treatment coverage (Africa region)	42.00% (NA)	https://www.who.int/data/gho/data/indicators/indicator-details/GHO/tuberculosis-effective-treatment-coverage-(-)
Malaria incidence	5,305,032.95 (2,941,398.38-8,422,177.01)	Institute for Health Metrics and Evaluation (IHME). GBD Compare. Seattle, WA: IHME, University of Washington, 2015. Available from http://vizhub.healthdata.org/gbd-compare. (Accessed [June 24, 2021])
Malaria incidence (Africa region)	212,519,605.86 (167,464,481.06-271,018,677.77)	Institute for Health Metrics and Evaluation (IHME). GBD Compare. Seattle, WA: IHME, University of Washington, 2015. Available

		from http://vizhub.healthdata.org/gbd-compare. (Accessed [June 24, 2021])
Malaria treatment cost per patient	$32.16 (3.43-67.22)	Sicuri, E., Vieta, A., Lindner, L. *et al.* The economic costs of malaria in children in three sub-Saharan countries: Ghana, Tanzania and Kenya. *Malar J* **12,** 307 (2013). https://doi.org/10.1186/1475-2875-12-307
Malaria drug cost per patient	$0.83 (NA)	Sicuri, E., Vieta, A., Lindner, L. *et al.* The economic costs of malaria in children in three sub-Saharan countries: Ghana, Tanzania and Kenya. *Malar J* **12,** 307 (2013). https://doi.org/10.1186/1475-2875-12-307
Malaria treatment coverage	80.00% (NA)	https://www.who.int/malaria/publications/country-profiles/profile_ken_en.pdf
Malaria treatment coverage (Africa region)	62.00% (NA)	https://data.unicef.org/topic/child-health/malaria/
Pneumonia incidence	3,044,843.06 (2,818,230.16-3,288,832.56)	Institute for Health Metrics and Evaluation (IHME). GBD Compare. Seattle, WA: IHME, University of Washington, 2015. Available from http://vizhub.healthdata.org/gbd-compare. (Accessed [June 24, 2021])

Pneumonia incidence (Africa region)	86,438,285.05 (80,114,956.08-93,353,097.24)	Institute for Health Metrics and Evaluation (IHME). GBD Compare. Seattle, WA: IHME, University of Washington, 2015. Available from http://vizhub.healthdata.org/gbd-compare. (Accessed [June 24, 2021])
Pneumonia treatment cost per patient	$143.13 (94.97-219.46)	Ayieko, P., Akumu, A. O., Griffiths, U. K., & English, M. (2009). The economic burden of inpatient paediatric care in Kenya: household and provider costs for treatment of pneumonia, malaria and meningitis. *Cost effectiveness and resource allocation : C/E*, *7*, 3. https://doi.org/10.1186/1478-7547-7-3
Pneumonia drug cost per patient	$13.00 (3.06-44.10)	Ayieko, P., Akumu, A. O., Griffiths, U. K., & English, M. (2009). The economic burden of inpatient paediatric care in Kenya: household and provider costs for treatment of pneumonia, malaria and meningitis. *Cost effectiveness and resource allocation : C/E*, *7*, 3. https://doi.org/10.1186/1478-7547-7-3
Pneumonia treatment coverage	61.00% (NA)	Knowles, R., Sharland, M., Hsia, Y., Magrini, N., Moja, L., Siyam, A., & Tayler, E. (2020). Measuring antibiotic availability and use in 20 low- and middle-income countries. *Bulletin of the World Health Organization*, *98*(3), 177–187C. https://doi.org/10.2471/BLT.19.241349
Pneumonia treatment coverage (Africa region)	65.00% (NA)	Knowles, R., Sharland, M., Hsia, Y., Magrini, N., Moja, L., Siyam, A., & Tayler, E. (2020). Measuring antibiotic availability and use in 20 low- and middle-income countries. *Bulletin of the World Health*

		Organization, *98*(3), 177–187C. https://doi.org/10.2471/BLT.19.241349
Diarrheal disease incidence	51,089,097.06 (2,818,230.16-3,288,832.56)	Institute for Health Metrics and Evaluation (IHME). GBD Compare. Seattle, WA: IHME, University of Washington, 2015. Available from http://vizhub.healthdata.org/gbd-compare. (Accessed [June 24, 2021])
Diarrheal disease incidence (Africa region)	1,490,211,697.73 (1,359,260,460.35-1,622,628,122.09)	Institute for Health Metrics and Evaluation (IHME). GBD Compare. Seattle, WA: IHME, University of Washington, 2015. Available from http://vizhub.healthdata.org/gbd-compare. (Accessed [June 24, 2021])
Diarrheal disease treatment cost per patient	$8.16 (NA)	Baral, R., Nonvignon, J., Debellut, F. *et al.* Cost of illness for childhood diarrhea in low- and middle-income countries: a systematic review of evidence and modelled estimates. *BMC Public Health* **20,** 619 (2020). https://doi.org/10.1186/s12889-020-08595-8
Diarrheal disease drug cost per patient	$3.90 (NA)	Baral, R., Nonvignon, J., Debellut, F. *et al.* Cost of illness for childhood diarrhea in low- and middle-income countries: a systematic review of evidence and modelled estimates. *BMC Public Health* **20,** 619 (2020). https://doi.org/10.1186/s12889-020-08595-8

Diarrheal disease treatment coverage	42.00% (38-47)	Lam, F., Wentworth, L., Cherutich, P., Migiro, S., Abdala, K., Musyoka, M., Ogolla, S., Obudho, M., Mwangi, Z., Kihoto, R., Cheruiyot, C., Wariari, B., Battu, A., & Schroder, K. (2019). An evaluation of a national oral rehydration solution and zinc scale-up program in Kenya between 2011 and 2016. *Journal of global health*, *9*(1), 010505. https://doi.org/10.7189/jogh.09.010505
Diarrheal disease treatment coverage (Africa region)	40.00% (NA)	Local Burden of Disease Diarrhoea Collaborators (2020). Mapping geographical inequalities in oral rehydration therapy coverage in low-income and middle-income countries, 2000-17. *The Lancet. Global health*, *8*(8), e1038–e1060. https://doi.org/10.1016/S2214-109X(20)30230-8
Employment rate	69.75% (NA)	https://ilostat.ilo.org/
Annual minimum wage	$2,199.17 (NA)	https://ilostat.ilo.org/
Domestic general government health expenditure (GGHE-D) as a percent of current health expenditure (CHE)	43.00% (NA)	https://apps.who.int/nha/database

Estimates and Data Sources used for South Africa Investment Case (Currencies Reported in 2021 US$)

Metric	Average Value (Range)	Source
Trial site start-up cost	$1,548,218.93 (NA)	https://www.idrc.ca/sites/default/files/sp/Documents%20EN/HIV-AIDS-Prevention-Trials-Capacity-Building-Grants-booklet.pdf
Trial site annual maintenance cost	$131,885.32 (103,214.60-137,619.46)	https://www.idrc.ca/sites/default/files/sp/Documents%20EN/HIV-AIDS-Prevention-Trials-Capacity-Building-Grants-booklet.pdf
Annual investment in training	$168,605.00 (157,848.31-178,170.76)	https://reporter.nih.gov/search/E522KYLLwUSNpwjPJQx64w/project-details/8319729<br><br>https://reporter.nih.gov/search/E522KYLLwUSNpwjPJQx64w/project-details/8319729<br><br>https://reporter.nih.gov/search/E522KYLLwUSNpwjPJQx64w/project-details/8319729<br><br>https://reporter.nih.gov/search/E522KYLLwUSNpwjPJQx64w/project-details/8319729<br><br>https://reporter.nih.gov/search/E522KYLLwUSNpwjPJQx64w/project-details/8319729

Vaccine phase 3 clinical trial cost	$122,210,000.00 (111,100,000.00-133,320,000.00)	Terry RF, Yamey G, Miyazaki-Krause R *et al.* Funding global health product R&D: the Portfolio-To-Impact Model (P2I), a new tool for modelling the impact of different research portfolios [version 2; peer review: 2 approved]. *Gates Open Res* 2018, **2**:24 (https://doi.org/10.12688/gatesopenres.12816.2)
Drug phase 3 clinical trial cost	$27,720,000.00 (17,610,000.00-59,530,000.00)	Terry RF, Yamey G, Miyazaki-Krause R *et al.* Funding global health product R&D: the Portfolio-To-Impact Model (P2I), a new tool for modelling the impact of different research portfolios [version 2; peer review: 2 approved]. *Gates Open Res* 2018, **2**:24 (https://doi.org/10.12688/gatesopenres.12816.2)
Trial site annual user fee	$1,118,851.30 (NA)	https://aspe.hhs.gov/report/examination-clinical-trial-costs-and-barriers-drug-development
Technology transfer royalties as a percentage of product sales	11.69% (1.25-48.80)	Borshell, N., Dawkes, A. Pharmaceutical royalties in licensing deals: No place for the 25 per cent rule of thumb. *J Commer Biotechnol* **16,** 8–16 (2010). https://doi.org/10.1057/jcb.2009.13
Product sale profits as a percentage of treatment costs	20.00% (NA)	https://www.mckinsey.com/industries/public-and-social-sector/our-insights/should-sub-saharan-africa-make-its-own-drugs

HIV disability weight	0.08 (0.01-0.58)	Global Burden of Disease Collaborative Network. Global Burden of Disease Study 2019 (GBD 2019) Disability Weights. Seattle, United States of America: Institute for Health Metrics and Evaluation (IHME), 2020.
TB disability weight	0.33 (0.33-0.33)	Global Burden of Disease Collaborative Network. Global Burden of Disease Study 2019 (GBD 2019) Disability Weights. Seattle, United States of America: Institute for Health Metrics and Evaluation (IHME), 2020.
Malaria disability weight	0.13 (0.01-0.40)	Global Burden of Disease Collaborative Network. Global Burden of Disease Study 2019 (GBD 2019) Disability Weights. Seattle, United States of America: Institute for Health Metrics and Evaluation (IHME), 2020.
Pneumonia disability weight	0.13 (0.13-0.13)	Global Burden of Disease Collaborative Network. Global Burden of Disease Study 2019 (GBD 2019) Disability Weights. Seattle, United States of America: Institute for Health Metrics and Evaluation (IHME), 2020.
Diarrheal disease disability weight	0.17 (0.07-0.25)	Global Burden of Disease Collaborative Network. Global Burden of Disease Study 2019 (GBD 2019) Disability Weights. Seattle, United States of America: Institute for Health Metrics and Evaluation (IHME), 2020.
HIV incidence	366,880.48 (286,119.75-464,312.35)	Institute for Health Metrics and Evaluation (IHME). GBD Compare. Seattle, WA: IHME, University of Washington, 2015. Available from http://vizhub.healthdata.org/gbd-compare. (Accessed [June 24, 2021])

HIV incidence (Africa region)	1,306,590.80 (1,093,181.64-1,576,684.33)	Institute for Health Metrics and Evaluation (IHME). GBD Compare. Seattle, WA: IHME, University of Washington, 2015. Available from http://vizhub.healthdata.org/gbd-compare. (Accessed [June 24, 2021])
HIV treatment cost per patient per year	$1,236.67 (575.75-2,258.72)	Meyer-Rath G, van Rensburg C, Chiu C, Leuner R, Jamieson L, Cohen S (2019) The per-patient costs of HIV services in South Africa: Systematic review and application in the South African HIV Investment Case. PLoS ONE 14(2): e0210497. https://doi.org/10.1371/journal.pone.0210497
HIV drug cost per patient per year	$52.16 (NA)	https://www.r4d.org/wp-content/uploads/South-Africa-HIV-and-TB-Expenditure-Review-2014-15-2016-17-Full-Report_vf.pdf?_ga=2.59364202.693959785.1621951931-765094531.1621951931
HIV treatment coverage	70.00% (64-74)	https://www.unaids.org/en/regionscountries/countries/southafrica
HIV treatment coverage (Africa region)	56.00% (NA)	https://aidsinfo.unaids.org/
TB incidence	219,127.09 (184,652.62-257,763.34)	Institute for Health Metrics and Evaluation (IHME). GBD Compare. Seattle, WA: IHME, University of Washington, 2015. Available from

		http://vizhub.healthdata.org/gbd-compare. (Accessed [June 24, 2021])
TB incidence (Africa region)	2,198,950.36 (1,921,392.55-2,525,066.04)	Institute for Health Metrics and Evaluation (IHME). GBD Compare. Seattle, WA: IHME, University of Washington, 2015. Available from http://vizhub.healthdata.org/gbd-compare. (Accessed [June 24, 2021])
TB treatment cost per patient	$2,013.99 (227.65-3,761.52)	van Rensburg C, Berhanu R, Hirasen K, Evans D, Rosen S, Long L (2019) Cost outcome analysis of decentralized care for drug-resistant tuberculosis in Johannesburg, South Africa. PLoS ONE 14(6): e0217820. https://doi.org/10.1371/journal.pone.0217820<br><br>Pooran, A., Pieterson, E., Davids, M., Theron, G., & Dheda, K. (2013). What is the cost of diagnosis and management of drug resistant tuberculosis in South Africa?. *PloS one*, *8*(1), e54587. https://doi.org/10.1371/journal.pone.0054587
TB drug cost per patient	$67.18 (19.92-160.89)	Pooran, A., Pieterson, E., Davids, M., Theron, G., & Dheda, K. (2013). What is the cost of diagnosis and management of drug resistant tuberculosis in South Africa?. *PloS one*, *8*(1), e54587. https://doi.org/10.1371/journal.pone.0054587

TB treatment coverage	59.00% (NA)	https://www.who.int/data/gho/data/indicators/indicator-details/GHO/tuberculosis-effective-treatment-coverage-(-)
TB treatment coverage (Africa region)	42.00% (NA)	https://www.who.int/data/gho/data/indicators/indicator-details/GHO/tuberculosis-effective-treatment-coverage-(-)
Malaria incidence	140,620.79 (4,347.55-903,673.73)	Institute for Health Metrics and Evaluation (IHME). GBD Compare. Seattle, WA: IHME, University of Washington, 2015. Available from http://vizhub.healthdata.org/gbd-compare. (Accessed [June 24, 2021])
Malaria incidence (Africa region)	212,519,605.86 (167,464,481.06-271,018,677.77)	Institute for Health Metrics and Evaluation (IHME). GBD Compare. Seattle, WA: IHME, University of Washington, 2015. Available from http://vizhub.healthdata.org/gbd-compare. (Accessed [June 24, 2021])
Malaria treatment cost per patient	$52.99 (10.41-165.20)	Wilkins, J. J., Folb, P. I., Valentine, N., & Barnes, K. I. (2002). An economic comparison of chloroquine and sulfadoxine-pyrimethamine as first-line treatment for malaria in South Africa: development of a model for estimating recurrent direct costs. *Transactions of the Royal Society of Tropical Medicine and Hygiene*, *96*(1), 85–90. https://doi.org/10.1016/s0035-9203(02)90251-8

		Muheki, C., McIntyre, D., & Barnes, K. I. (2004). Artemisinin-based combination therapy reduces expenditure on malaria treatment in KwaZulu Natal, South Africa. *Tropical medicine & international health : TM & IH*, *9*(9), 959–966. https://doi.org/10.1111/j.1365-3156.2004.01292.x
Malaria drug cost per patient	$3.93 (2.44-5.28)	Wilkins, J. J., Folb, P. I., Valentine, N., & Barnes, K. I. (2002). An economic comparison of chloroquine and sulfadoxine-pyrimethamine as first-line treatment for malaria in South Africa: development of a model for estimating recurrent direct costs. *Transactions of the Royal Society of Tropical Medicine and Hygiene*, *96*(1), 85–90. https://doi.org/10.1016/s0035-9203(02)90251-8<br><br>Muheki, C., McIntyre, D., & Barnes, K. I. (2004). Artemisinin-based combination therapy reduces expenditure on malaria treatment in KwaZulu Natal, South Africa. *Tropical medicine & international health : TM & IH*, *9*(9), 959–966. https://doi.org/10.1111/j.1365-3156.2004.01292.x
Malaria treatment coverage	80.00% (NA)	https://www.who.int/malaria/publications/country-profiles/profile_zaf_en.pdf?ua=1
Malaria treatment coverage (Africa region)	62.00% (NA)	https://data.unicef.org/topic/child-health/malaria/

Pneumonia incidence	4,835,818.09 (4,516,636.91-5,157,559.73)	Institute for Health Metrics and Evaluation (IHME). GBD Compare. Seattle, WA: IHME, University of Washington, 2015. Available from http://vizhub.healthdata.org/gbd-compare. (Accessed [June 24, 2021])
Pneumonia incidence (Africa region)	86,438,285.05 (80,114,956.08-93,353,097.24)	Institute for Health Metrics and Evaluation (IHME). GBD Compare. Seattle, WA: IHME, University of Washington, 2015. Available from http://vizhub.healthdata.org/gbd-compare. (Accessed [June 24, 2021])
Pneumonia treatment cost per patient	$516.21 (NA)	Kitchin, O. P., Wessels, F., Masekela, R., Becker, P., & Green, R. J. (2011). Costs of admission for paediatric pneumonia in a setting of human immunodeficiency virus infection. *The international journal of tuberculosis and lung disease : the official journal of the International Union against Tuberculosis and Lung Disease*, *15*(12), 1702–1707. https://doi.org/10.5588/ijtld.11.0167
Pneumonia drug cost per patient	$19.90 (19.00-20.79)	Kitchin, O. P., Wessels, F., Masekela, R., Becker, P., & Green, R. J. (2011). Costs of admission for paediatric pneumonia in a setting of human immunodeficiency virus infection. *The international journal of tuberculosis and lung disease : the official journal of the International Union against Tuberculosis and Lung Disease*, *15*(12), 1702–1707. https://doi.org/10.5588/ijtld.11.0167
Pneumonia treatment coverage	52.00% (NA)	https://data.unicef.org/topic/child-health/pneumonia/

Pneumonia treatment coverage (Africa region)	65.00% (NA)	Knowles, R., Sharland, M., Hsia, Y., Magrini, N., Moja, L., Siyam, A., & Tayler, E. (2020). Measuring antibiotic availability and use in 20 low- and middle-income countries. *Bulletin of the World Health Organization*, *98*(3), 177–187C. https://doi.org/10.2471/BLT.19.241349
Diarrheal disease incidence	48,757,253.51 (44,772,598.90-53,274,520.78)	Institute for Health Metrics and Evaluation (IHME). GBD Compare. Seattle, WA: IHME, University of Washington, 2015. Available from http://vizhub.healthdata.org/gbd-compare. (Accessed [June 24, 2021])
Diarrheal disease incidence (Africa region)	1,490,211,697.73 (1,359,260,460.35-1,622,628,122.09)	Institute for Health Metrics and Evaluation (IHME). GBD Compare. Seattle, WA: IHME, University of Washington, 2015. Available from http://vizhub.healthdata.org/gbd-compare. (Accessed [June 24, 2021])
Diarrheal disease treatment cost per patient	$24.26 (NA)	Baral, R., Nonvignon, J., Debellut, F. *et al.* Cost of illness for childhood diarrhea in low- and middle-income countries: a systematic review of evidence and modelled estimates. *BMC Public Health* **20,** 619 (2020). https://doi.org/10.1186/s12889-020-08595-8
Diarrheal disease drug cost per patient	$3.90 (NA)	Baral, R., Nonvignon, J., Debellut, F. *et al.* Cost of illness for childhood diarrhea in low- and middle-income countries: a systematic review of

		evidence and modelled estimates. *BMC Public Health* **20,** 619 (2020). https://doi.org/10.1186/s12889-020-08595-8
Diarrheal disease treatment coverage	47.00% (NA)	Chola, L., Michalow, J., Tugendhaft, A., & Hofman, K. (2015). Reducing diarrhoea deaths in South Africa: costs and effects of scaling up essential interventions to prevent and treat diarrhoea in under-five children. *BMC public health*, *15*, 394. https://doi.org/10.1186/s12889-015-1689-2
Diarrheal disease treatment coverage (Africa region)	40.00% (NA)	Local Burden of Disease Diarrhoea Collaborators (2020). Mapping geographical inequalities in oral rehydration therapy coverage in low-income and middle-income countries, 2000-17. *The Lancet. Global health*, *8*(8), e1038–e1060. https://doi.org/10.1016/S2214-109X(20)30230-8
Employment rate	36.69% (NA)	https://ilostat.ilo.org/
Annual minimum wage	$3023.16 (NA)	https://ilostat.ilo.org/
Domestic general government health expenditure (GGHE-D) as a percent of current health expenditure (CHE)	57.00% (NA)	https://apps.who.int/nha/database

Regulation Estimates and Data Sources for India, South Africa, and Kenya Investment Cases (Currencies Reported in 2021 US$)

Metric	Average Value (Range)	Source
Pharmaceutical market size of top 20 national pharmaceutical markets in the world.	$54,680,080,000.00 (6,861,400,000-527,800,000,000)	IQVIA Institute for Human Data Science. Global medicine spending and usage trends, outlook to 2025. https://www.iqvia.com/insights/the-iqvia-institute/reports/global-medicine-spending-and-usage-trends-outlook-to-2025
Pharmaceutical market size of India	$21,112,000,000 (NA)	QVIA Institute for Human Data Science. Global medicine spending and usage trends, outlook to 2025. https://www.iqvia.com/insights/the-iqvia-institute/reports/global-medicine-spending-and-usage-trends-outlook-to-2025
Pharmaceutical market size of Kenya	$750,000,000 (NA)	https://www.asokoinsight.com/content/quick-insights/kenya-leading-pharmaceutical-companies
Pharmaceutical market size of South Africa	$3,400,000,000 (NA)	Africa Health. Industry insights: South Africa healthcare market overview. (2020). https://www.africahealthexhibition.com/en/overview/industry-insights/south-africa-healthcare-market-overview/page-3.html
Annual spending on regulation in United States	$5,798,555,000 (NA)	https://www.fda.gov/about-fda/budgets/2019-fda-justification-estimates-appropriations-committees-browse-section
Annual spending on regulation in Japan	$288,468,149 (NA)	https://www.pmda.go.jp/english/about-pmda/annual-reports/0001.html

Annual spending on regulation in France	$147,154,600 (NA)	https://ansm.sante.fr/qui-sommes-nous/publications-institutionnelles/publications-2019
Annual spending on regulation in United Kingdom	$204,688,480 (NA)	https://www.gov.uk/government/publications/medicines-and-healthcare-products-regulatory-agency-annual-report-and-accounts-2019-to-2020
Annual spending on regulation in Spain	$57,452,352 (NA)	https://www.aemps.gob.es/laAEMPS/memoria/docs/memoria-2019.pdf?x16990
Annual spending on regulation in Brazil	$231,223,951 (NA)	http://www.portaldatransparencia.gov.br/download-de-dados/orcamento-despesa
Annual spending on regulation in Canada	$524,343,395 (NA)	https://www.canada.ca/en/treasury-board-secretariat/services/planned-government-spending/government-expenditure-plan-main-estimates/2018-19-estimates/2018-19-budgetary-expenditures-strategic-outcome-program.html
Annual spending on regulation in India	$269,019,648 (NA)	https://openbudgetsindia.org/dataset?tags=central+drugs+standard+control+organisation
Annual spending on regulation in Australia	$170,000,000 (NA)	https://www.tga.gov.au/sites/default/files/tga-business-plan-2020-21.pdf
Annual spending on regulation in Turkey	$28,000,000 (NA)	https://www.frontiersin.org/articles/10.3389/fphar.2019.01557/full#B12

Annual spending on regulation in Belgium	$88,478,555 (NA)	https://www.famhp.be/sites/default/files/content/ar_2017_complet.pdf
Annual spending on regulation in South Africa	$16,342,832 (NA)	https://www.sahpra.org.za/wp-content/uploads/2020/12/SAHPRA-ANNUAL-REPORT-2020-web.pdf
Annual spending on regulation in Kenya	$5,722,668 (NA)	Kenya National Audit Office. Report of the Auditor General on the Financial Statements of the Pharmacy and Poisons Board. (2014).

Annex 4: Model equations

Average years of life lost per death

$$YLL = \frac{\sum_{a=1}^{5}(D_a * L_a)}{D} \qquad \text{Equation 1}$$

Where YLL is the average years of life lost per death, a is age group, D is number of deaths per year, and L is life expectancy.

Average years of life lost to disability per non-treated case

$$YLD = \frac{\sum_{a=1}^{5}(I_a * T_a * DW)}{I} \qquad \text{Equation 2}$$

Where YLD is the average years of life lost to disability per non-treated case, a is age group, I is annual incidence, T is disease duration without treatment expressed in years, DW is the disability weight. All disability weights were collected from the IHME database.

Average years of life lost to disability per treated case

$$YLD = \frac{\sum_{a=1}^{5}(I_a * T_a * DW)}{I} \qquad \text{Equation 3}$$

Where YLD is the average years of life lost to disability per treated case, a is age group, I is annual incidence, T is disease duration with treatment expressed in years, DW is the disability weight. All disability weights were collected from the IHME database.

Number of cases averted

$$N = \sum_{i=1}^{5}\sum_{x=1}^{16}(IB_{i,x} - IV_{i,x}) \qquad \text{Equation 4}$$

Where N is the number of cases averted, i is the disease, x is the year, IB is the baseline incidence of disease, and IV is the incidence of disease with a new vaccine.

Number of deaths averted

$$N = \sum_{i=1}^{5}\sum_{x=1}^{16}\left[\left(IB_{i,x} * \left(1 - CB_{i,x}\right) * CFR_i\right) + \left(IB_{i,x} * \left(CB_{i,x}\right) * CFRT_i\right)\right] - \left[\left(IV_{i,x} * \left(1 - CD_{i,x}\right) * CFR_i\right) + \left(IV_{i,x} * \left(CD_{i,x}\right) * CFRT_i\right)\right] \qquad \text{Equation 5}$$

Where N is the number of deaths averted, i is the disease, x is the year, IB is the baseline incidence, CB is the baseline treatment coverage, CFR is the case fatality rate without treatment, CFRT is the case fatality rate with treatment, IV is the incidence with a new vaccine, and CD is the treatment coverage with a new drug.

Number of years of life lost to death averted

$$N = \sum_{i=1}^{5} \sum_{x=1}^{16} (DB_{i,x} * YLL_i - DT_{i,x} * YLL_i) \quad \text{Equation 6}$$

Where N is the number of years of life lost to death averted, i is the disease, x is the year, DB is the baseline number of deaths, YLL is the average years of life lost per death, and DT is the number of deaths with a new vaccine and drug.

Number of years of life lost to disability averted

$$N = \sum_{i=1}^{5} \sum_{x=1}^{16} \left[\left(IB_{i,x} * \left(1 - CB_{i,x}\right) * YLD_i \right) + \left(IB_{i,x} * \left(CB_{i,x}\right) * YLDT_i \right) \right] - \left[\left(IV_{i,x} * \left(1 - CD_{i,x}\right) * YLD_i \right) + \left(IV_{i,x} * \left(CD_{i,x}\right) * YLDT_i \right) \right] \quad \text{Equation 7}$$

Where N is the number of years of life lost to disability averted, i is the disease, x is the year, IB is the baseline incidence, CB is the baseline treatment coverage, YLD is the average number of years of life lost to disability per non-treated case, YLDT is the average number of years of life lost to disability per treated case, IV is the incidence with a new vaccine, and CD is the treatment coverage with a new drug.

Number of disability adjusted life years averted

$$N = \sum_{i=1}^{5} \sum_{x=1}^{16} (YLL_{i,x} + YLD_{i,x}) \quad \text{Equation 8}$$

Where N is the number of disability adjusted life years averted, i is the disease, x is the year, YLL is the number of years of life lost to death averted, and YLD is the number of years of life lost to disability averted.

Treatment costs averted

$$C = \sum_{i=1}^{5} \sum_{x=1}^{16} (N_{i,x} * K_{i,x}) \quad \text{Equation 9}$$

Where C is treatment costs averted, i is the disease, x is the year, N is the number of cases averted, and K is the cost per case treated.

Number of vaccine doses needed

$$N = \frac{\sum_{i=1}^{5} \sum_{x=1}^{16} (A_{i,x})}{E} \quad \text{Equation 10}$$

Where N is number of vaccine doses needed, i is the disease, x is the year, A is the number of cases averted, and E is vaccine efficacy.

Vaccine procurement costs

$$C = \sum_{i=1}^{5} \sum_{x=1}^{16} (N_{i,x} * K_{i,x}) \qquad \text{Equation 11}$$

Where C is vaccine procurement costs, i is the disease, x is the year, N is the number of vaccine doses needed, K is the procurement cost per vaccine dose.

Number of new cases treated

$$N = \sum_{i=1}^{5} \sum_{x=1}^{16} (IV_{i,x} * CD_{i,x} - IB_{i,x} * CB_{i,x}) \qquad \text{Equation 12}$$

Where N is number of new cases treated, i is the disease, x is the year, IV is the incidence with a new vaccine, CD is the treatment coverage with a new drug, IB is the baseline incidence, CB is the baseline treatment coverage.

Cost of new cases treated

$$C = \sum_{i=1}^{5} \sum_{x=1}^{16} (N_{i,x} * K_{i,x}) \qquad \text{Equation 13}$$

Where C is cost of new cases treated, i is the disease, x is the year, N is the number of new cases treated, K is the cost per case treated.

Clinical trial site startup costs

$$C = \sum_{i=1}^{5} \sum_{x=1}^{16} (T_{i,x} * K_{i,x}) \qquad \text{Equation 14}$$

Where C is clinical trial site start-up costs, i is the disease, x is the year, T is the number of new clinical trial sites started, and K is the startup cost per clinical trial site.

Clinical trial site maintenance costs

$$C = \sum_{i=1}^{5} \sum_{x=1}^{16} (T_{i,x} * K_{i,x}) \qquad \text{Equation 15}$$

Where C is clinical trial site maintenance costs, i is disease, x is year, T is the number of new clinical trial sites in existence, and K is the annual cost to maintain one clinical trial site.

Late-stage clinical trial costs

$$C = \sum_{i=1}^{5} \sum_{x=1}^{16} (T_{i,x} * K_{i,x}) \qquad \text{Equation 16}$$

Where C is late-stage clinical trial costs, i is the disease, x is the year, T is the number of clinical trials started, and K is the cost per phase 3 clinical trial.

Clinical trial training costs

$$C = \sum_{x=1}^{16}(K_x) \quad \text{Equation 17}$$

Where C is clinical trial training costs, x is the year, and K is the annual investment in clinical trial training.

Returns from trial site user fees

$$R = \sum_{i=1}^{5}\sum_{x=1}^{16}(T_{i,x} * C_{i,x}) \quad \text{Equation 18}$$

Where R is returns from trial site user fees, i is the disease, x is the year, T is the number of clinical trial sites in use, and C is the annual cost to use a clinical trial site.

Returns from product sales

$$R = \sum_{i=1}^{5}\sum_{x=1}^{16}\left(V_{i,x} * CV_{i,x} + D_{i,x} * CD_{i,x}\right) * M \quad \text{Equation 19}$$

Where R is returns from product sales, i is the disease, x is the year, V is the number of vaccines manufactured nationally, CV is the cost per vaccine dose, D is the number of drugs manufactured nationally, CD is the cost per drug dose, and M is the profit margin on vaccine and drug sales.

Returns from tech transfer

$$R = \sum_{i=1}^{5}\sum_{x=1}^{16}\left(V_{i,x} * CV_{i,x} + D_{i,x} * CD_{i,x}\right) * M * Y \quad \text{Equation 20}$$

Where R is returns from tech transfer, i is the disease, x is the year, V is the number of vaccines manufactured through tech transfer, CV is the cost per vaccine dose, D is the number of drugs manufactured through tech transfer, CD is the cost per drug dose, M is the profit margin on vaccine and drug sales, and Y is the royalty rate on the sale vaccines and drugs manufactured through tech transfer.

Investment in health regulation

$$K = \sum_{x=1}^{16}(R * S) - A \quad \text{Equation 21}$$

Where K is the investment in health regulation, x is the year, R is the average ratio of pharmaceutical regulation spending to total pharmaceutical market size of the countries with the top ten largest pharmaceutical markets, S is the current pharmaceutical market size of the country of interest, and A is current spending on pharmaceutical regulation in the country of interest.

Economic productivity

$$E = \sum_{i=1}^{5} \sum_{x=1}^{16} [(YLL_{i,x} * D_{i,x} * R * W) + (YLD_{i,x} * C_{i,x} * R * W)] \quad \text{Equation 22}$$

Where E is total economic productivity, i is the disease, x is the year, YLL is the years of life lost to death averted, YLD is the years of life lost to disability averted, D is the percent of deaths age 15 to 69, C is the percent of cases age 15 to 69, R is the employment rate, and W is the minimum wage.

Annex 5: Inputs from P2I model

	**Cost per phase (US$ millions)**				**Length of phase (years)**				**Probability of success**			
**Archetype**	Preclinical	Phase 1	Phase 2	Phase 3	Preclinical	Phase 1	Phase 2	Phase 3	Preclinical	Phase 1	Phase 2	Phase 3
Vaccine-simple	$6.66	$2.25	$13.22	$111.10	3.36	1.57	2.23	2.33	41.0%	68.4%	45.9%	70.8%
Vaccine-complex	$16.63	$2.47	$13.88	$133.32	3.33	1.97	3.71	3.50	41.0%	50.0%	21.6%	63.6%
NCE-simple	$5.00	$2.21	$5.81	$32.82	2.49	1.80	3.38	3.18	65.0%	59.7%	38.8%	69.1%
NCE-innovative	$7.50	$4.83	$6.10	$34.46	2.70	1.81	3.35	3.10	60.0%	51.9%	28.4%	57.8%
NCE-complex	$10.00	$7.44	$6.39	$36.10	2.87	1.93	3.51	2.80	55.0%	57.2%	19.7%	40.3%
Drug repurpose-simple	$0.00	$0.00	$5.81	$17.61	0.00	0.00	2.14	2.14	100.0%	100.0%	45.7%	68.1%
Drug repurpose-complex	$5.00	$2.21	$5.81	$17.61	2.33	1.63	2.14	2.14	75.0%	58.5%	45.7%	68.1%
Biologic-simple	$10.79	$2.41	$7.53	$54.12	3.29	1.62	2.47	2.10	75.0%	66.2%	44.3%	70.9%
Biologic-complex	$21.59	$7.65	$8.28	$59.53	3.24	1.49	4.16	3.38	77.0%	69.6%	32.2%	62.5%
Diagnostic-assay dev.	$3.00	$2.00	$3.50	$0.00	1.00	1.25	1.33	0.00	50.0%	100.0%	100.0%	100.0%
Diagnostic-simple platform dev.	$0.00	$100.00	$3.50	$0.00	0.00	2.50	2.00	0.00	100.0%	75.0%	100.0%	100.0%

Annex 6. Sensitivity analysis results (BCRs), US$2021

We conducted six sensitivity analyses (SAs) to account for uncertainty in our parameter estimates. The standard 3.0% discount rate used in global health economic evaluations may be inconsistent with low- and middle-income economies, so we increased the discount rate to 5.0% (SA 1).[1] We also increased the proportion of phase III clinical trial costs and manufacturing costs covered by countries from 10% to 20% due to a limited availability of data regarding government cost contributions to clinical trials and manufacturing (SA 2). To further account for limited data on government costs contributions to clinical trials and manufacturing, we conducted an additional sensitivity analysis where we increased government coverage of phase III trial costs and manufacturing costs from 10% to 50% (SA 3). To account for potentially low estimates of phase III clinical trial costs presented in the P2I model, we increased all phase III trial costs by 10% (SA 4). We also reduced the P2I phase III transition probabilities from average to minimum values to account for potential inefficiencies in clinical trial designs (SA 5).

Lastly, while the major focus of our study was on the direct financial gains that result from investments in clinical trial and manufacturing, we also added economic productivity to the societal perspective, to capture the longer-term benefits of these investments (SA 6). Economic productivity was calculated by monetizing YLLs averted and YLDs averted (Annex 3, Equation 22). Economic productivity from YLLs averted was calculated as the product of total YLLs averted among the working population and annual minimum wage. Similarly, economic productivity from YLDs averted was calculated as the product of total YLDs averted among the working population and annual minimum wage. Annual minimum wage estimates and employment rates used to define the working population can be found in Annex 2.

Country	Scenario	Perspective	Original BCR	SA 1	SA 2	SA 3	SA 4	SA 5	SA 6
India	Domestic	Country	62.42	51.67	38.14	17.60	59.30	54.54	/
		Societal	27.82	22.14	27.82	27.82	25.80	25.39	46.59
	Regional	Country	60.71	50.28	37.10	17.12	57.67	53.05	/
		Societal	66.56	53.97	66.56	66.56	61.73	60.73	88.28
Kenya	Domestic	Country	2.51	2.11	1.50	0.68	2.38	2.29	/
		Societal	0.73	0.59	0.73	0.73	0.68	0.68	9.69
	Regional	Country	8.78	7.54	5.25	2.38	8.32	7.87	/
		Societal	20.51	16.86	20.51	20.51	19.01	18.78	100.16
South Africa	Domestic	Country	7.01	6.03	5.09	2.79	6.80	6.32	/
		Societal	2.85	2.32	2.85	2.85	2.66	2.41	27.64
	Regional	Country	11.88	10.47	8.62	4.73	11.52	10.69	/

		Societal	33.27	27.63	33.27	33.27	31.06	28.07	130.86

SA 1 = discount rate changed from 3.0% to 5.0%

SA 2 = countries cover 20% of phase III clinical trial costs and manufacturing capacity building costs.

SA 3 = countries cover 50% of phase III clinical trial costs and manufacturing capacity building costs.

SA 4 = phase III clinical trial costs increased by 10%.

SA 5 = phase III clinical trial transition probabilities changed to minimum values instead of average values.

SA 6 = adds monetization of DALYs (using minimum wage and mean employment) as a benefit in the societal perspective.

1. Haacker M, Hallett TB, Atun R. On discount rates for economic evaluations in global health. Health Policy Plan. 2020 Feb 1;35(1):107–14.

Annex 7. Health and economic benefits stratified by disease

Health Benefits

Our results show that the health benefits of investments in clinical trial and manufacturing capacity are not distributed evenly across the five diseases of interest. In India's domestic scenario, for example, pneumonia accounts for 53.2%, TB 22.1%, diarrhea 19.4%, HIV 3.7%, and malaria 1.6% of all disability-adjusted life years (DALYs) averted. India's regional scenario has a similar distribution of DALYs averted. In Kenya's domestic scenario, HIV accounts for 40.4%, pneumonia 18.1%, malaria 17.9%, TB 14.3%, and diarrhea 9.3% of all DALYs averted. In Kenya's regional scenario, malaria accounts for 29.3%, HIV 26.1%, pneumonia 20.9%, TB 13.1%, and diarrhea 10.6% of all DALYs averted. In South Africa's domestic scenario, HIV accounts for 78.5%, pneumonia 9.7%, TB 8.5%, diarrhea 3.1%, and malaria 0.2% of all DALYs averted. In South Africa's regional scenario, HIV accounts for 53.6%, malaria 21.0%, pneumonia 10.9%, TB 9.6%, and diarrhea 4.9% of all DALYs averted. Generally, the magnitude of DALYs averted for each disease reflects both the national disease burden, health seeking behaviors in each country, and the disability weights of each disease. In India, for example, the incidence of pneumonia is much greater than HIV, TB, and malaria, but less than the incidence of diarrhea. However, pneumonia has a higher number of YLDs per case than diarrhea. Consequently, pneumonia accounts for a majority of the DALYs averted in India. Similarly, in South Africa, the incidence of HIV is slightly more than that of TB and malaria, but much less than the incidence of pneumonia and diarrhea. However, HIV has a much larger number of YLDs per case than pneumonia and diarrhea. Consequently, HIV accounts for a majority of DALYs averted in South Africa.

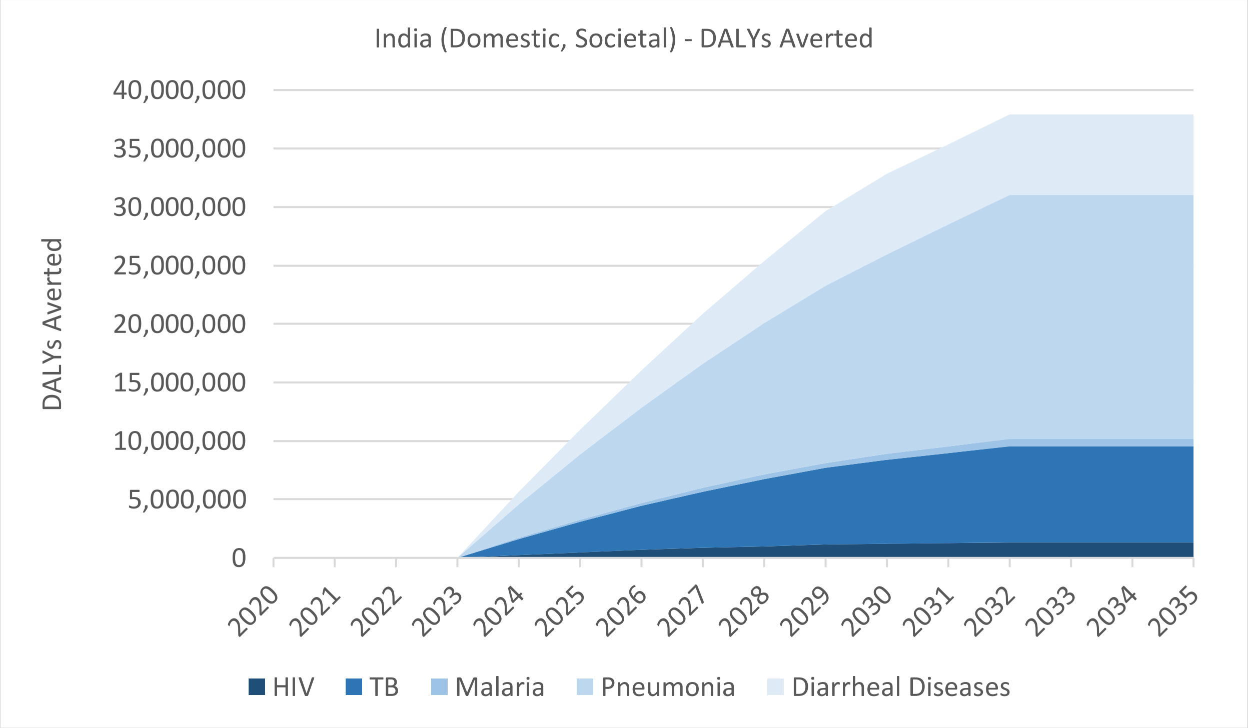

Figure A1. DALYs averted stratified by disease from the India domestic, societal perspective. The DALYs averted in any given year are cumulative across diseases, but not across previous years.

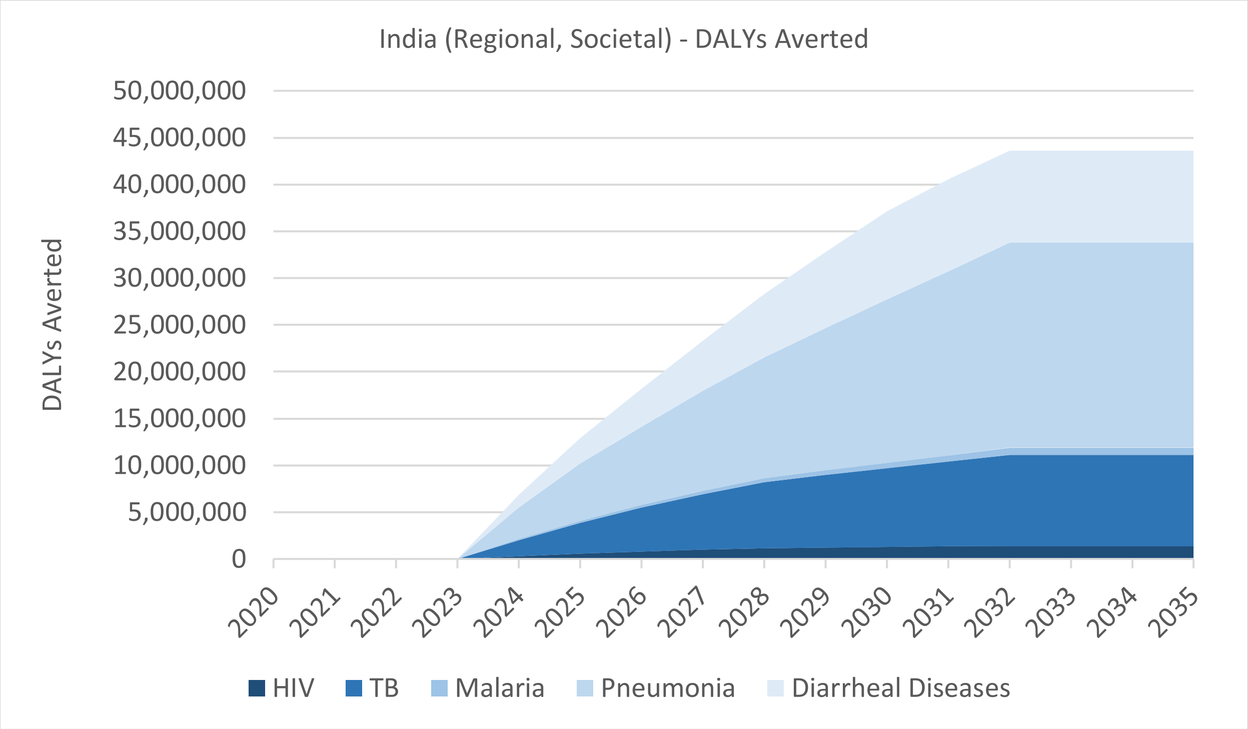

Figure A2. DALYs averted stratified by disease from the India regional, societal perspective. The DALYs averted in any given year are cumulative across diseases, but not across previous years.

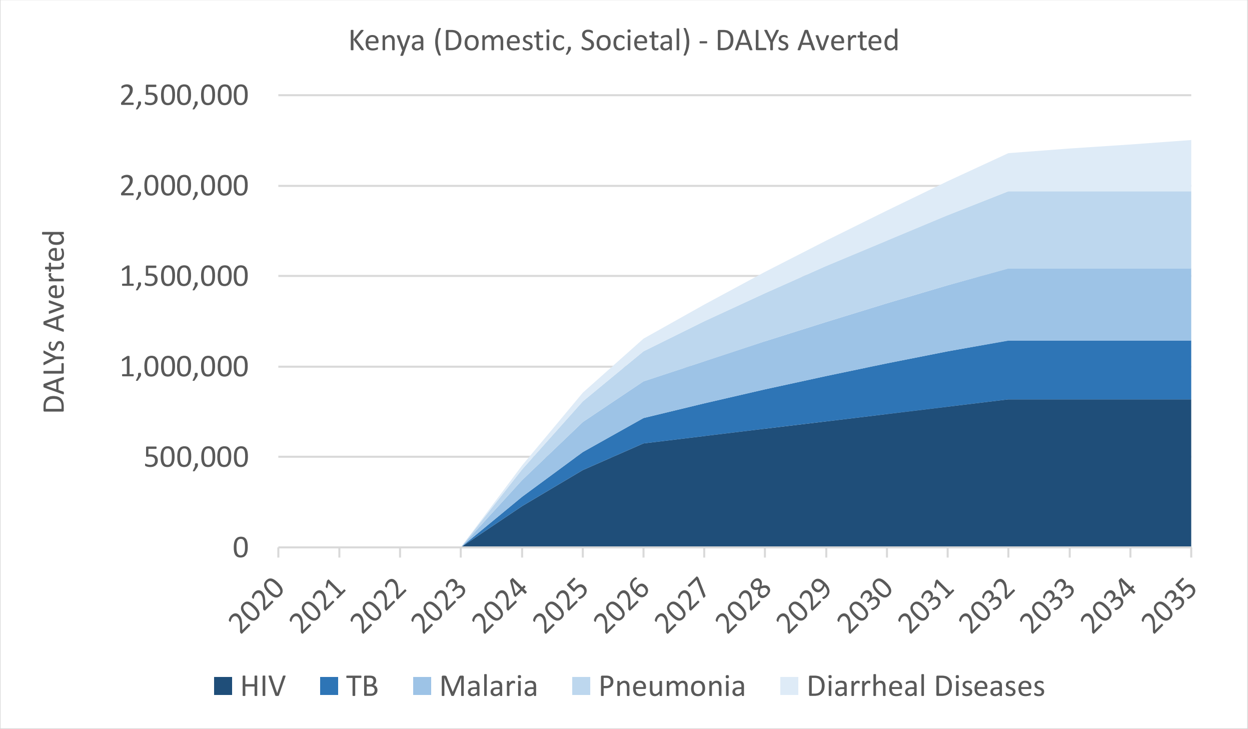

Figure A3. DALYs averted stratified by disease from the Kenya domestic, societal perspective. The DALYs averted in any given year are cumulative across diseases, but not across previous years.

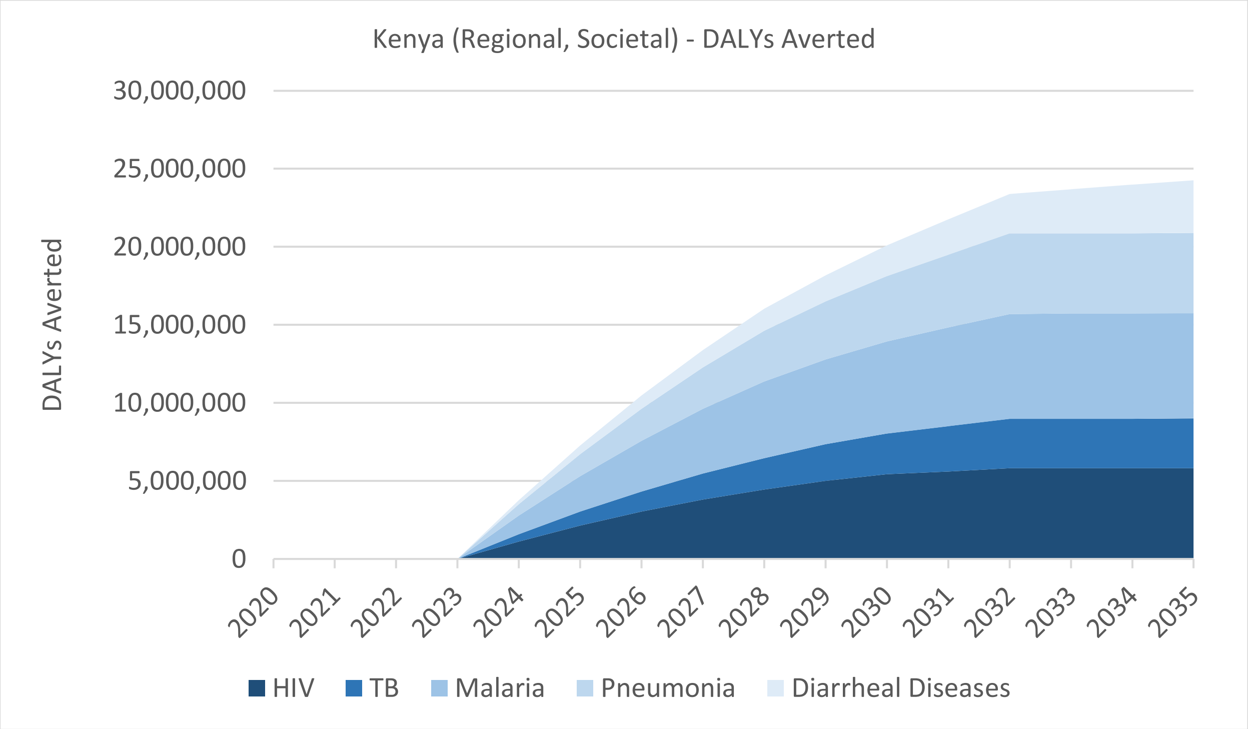

Figure A4. DALYs averted stratified by disease from the Kenya regional, societal perspective. The DALYs averted in any given year are cumulative across diseases, but not across previous years.

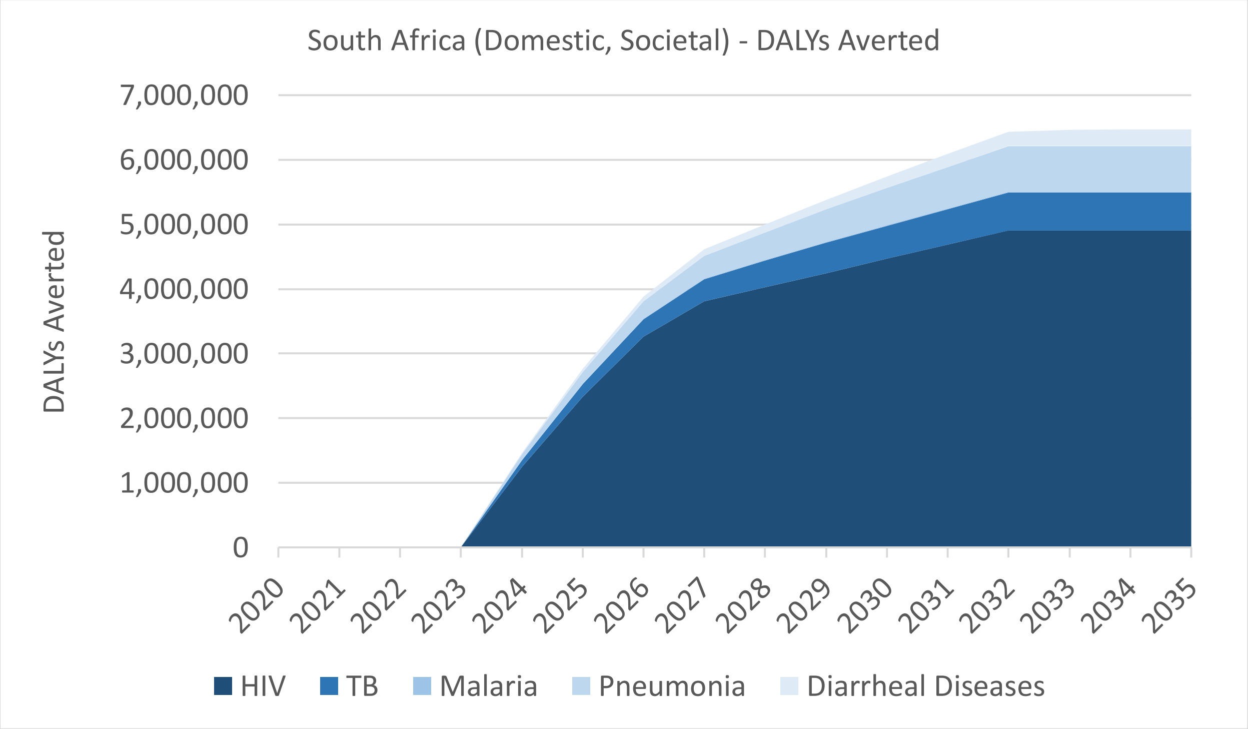

Figure A5. DALYs averted stratified by disease from the South Africa domestic, societal perspective. The DALYs averted in any given year are cumulative across diseases, but not across previous years.

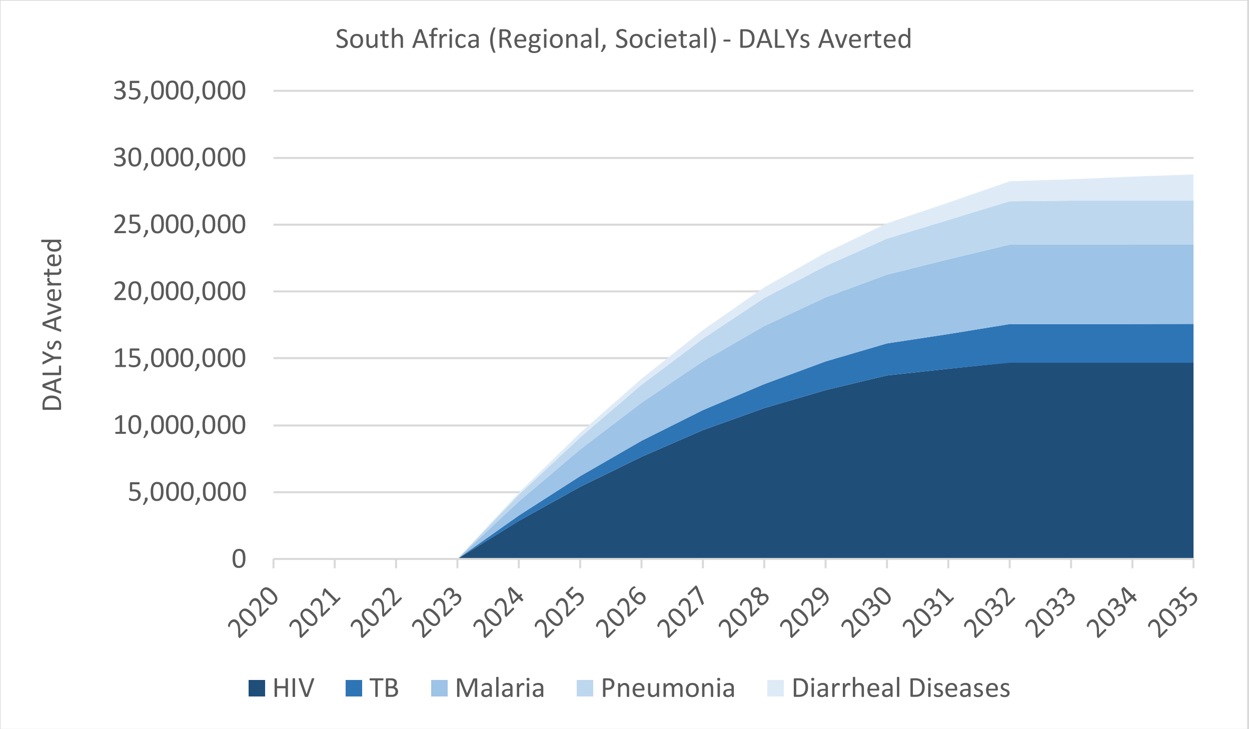

Figure A6. DALYs averted stratified by disease from the South Africa regional, societal perspective. The DALYs averted in any given year are cumulative across diseases, but not across previous years.

Economic Benefits

Our results show that the economic benefits of investments in clinical trial and manufacturing capacity are not distributed evenly across the diseases. In our model, averted treatment costs are a product of averted cases. Additionally, cases are only averted through new vaccines and not new drugs. Since we do not model a new vaccine for diarrheal diseases, there are no averted cases of diarrhea and therefore no averted treatment costs for diarrhea.

In India's domestic scenario, for example, pneumonia accounts for 82.6%, TB 15.6%, malaria 1.6%, and HIV 0.2% of all treatment costs averted. India's regional scenario has a similar distribution of treatment costs averted across diseases. In Kenya's domestic scenario, pneumonia accounts for 46.3%, TB 27.2%, malaria 23.8%, and HIV 2.7% of all treatment costs averted. The distribution is similar in the regional scenario. In South Africa's domestic scenario, pneumonia accounts for 69.1%, HIV 16.9%, TB 13.7%, and malaria 0.3% of all treatment costs averted. In South Africa's regional scenario pneumonia accounts for 70.7%, malaria 17.5%, TB 6.8%, and HIV 5.0% of all treatment costs averted. Generally, the magnitude of treatment costs averted through investments in each disease reflect the disease burden in each country. For example, pneumonia's large contribution to treatment costs averted is a result of both its high cost of treatment and high incidence in all three countries, relative to the other diseases. Similarly, in South Africa where the burden of HIV is the highest in the world, HIV is a large contributor to treatment costs averted in the domestic scenario, but the smallest contributor to treatment costs averted in the regional scenario as the burden of HIV in surrounding countries in comparatively small.

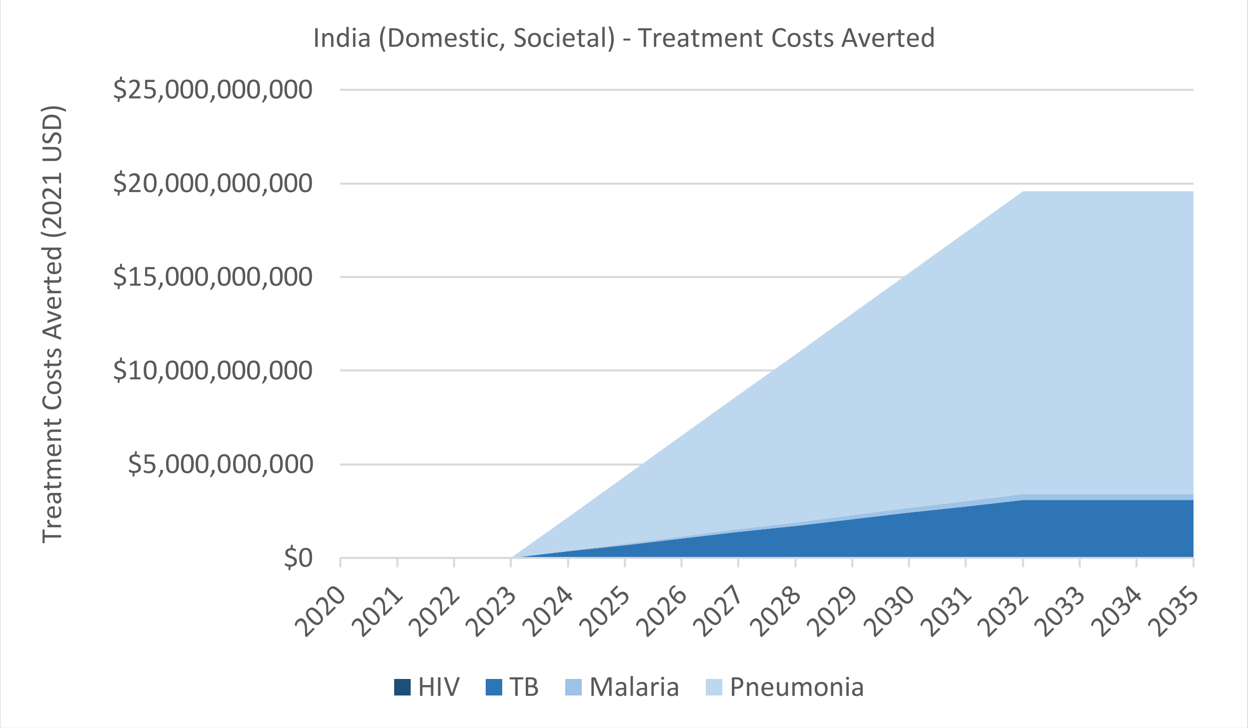

Figure A7. Treatment costs averted stratified by disease from the India domestic, societal perspective. The treatment costs averted in any given year are cumulative across diseases, but not across previous years.

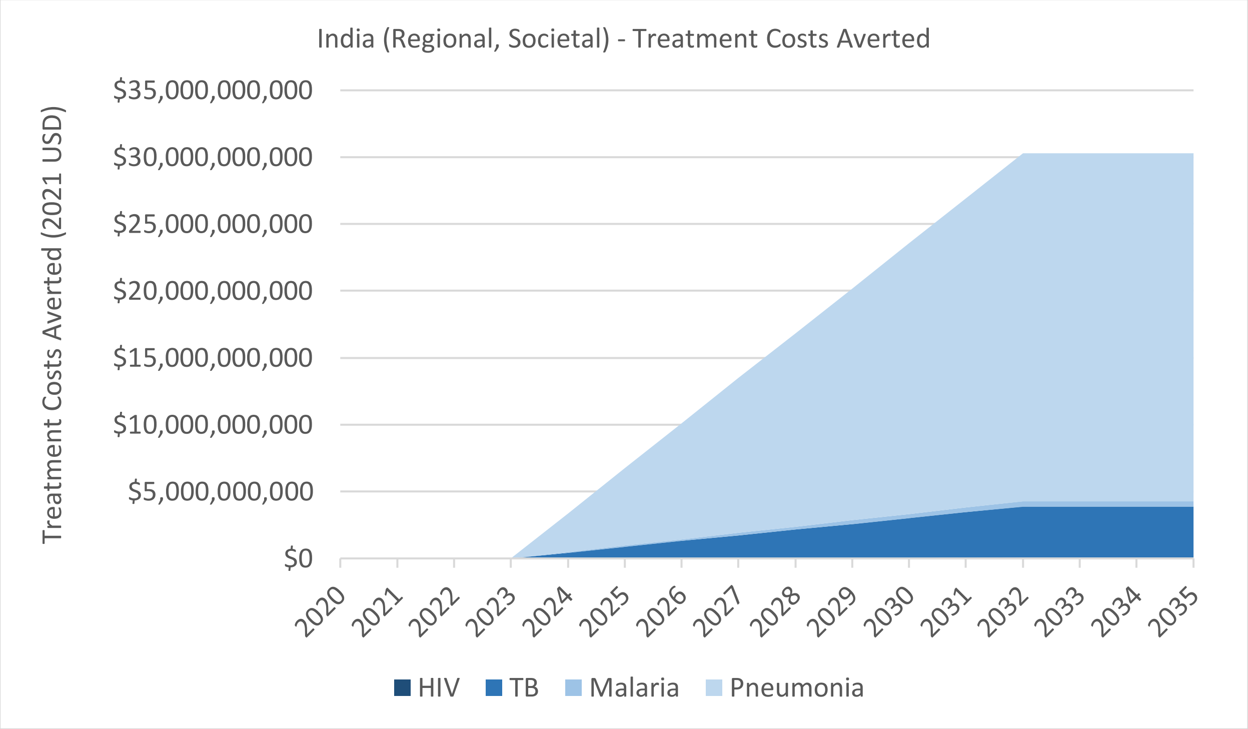

Figure A8. Treatment costs averted stratified by disease from the India regional, societal perspective. The treatment costs averted in any given year are cumulative across diseases, but not across previous years.

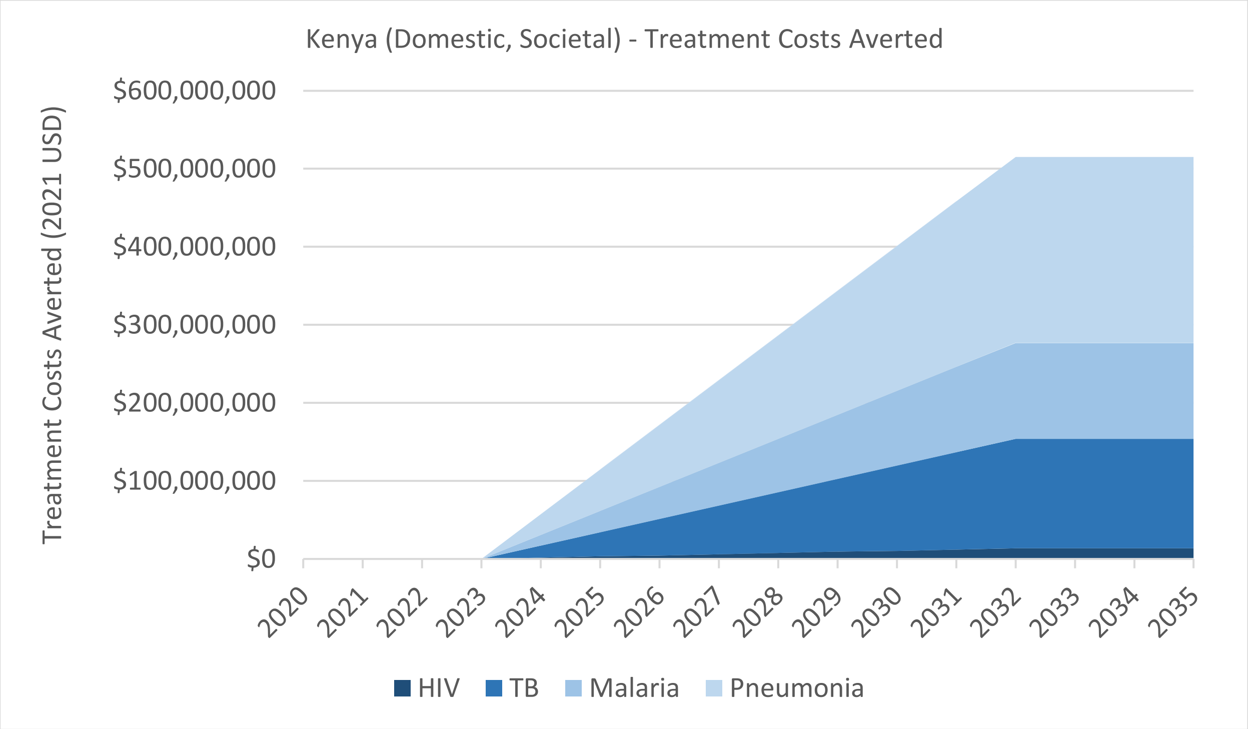

Figure A9. Treatment costs averted stratified by disease from the Kenya domestic, societal perspective. The treatment costs averted in any given year are cumulative across diseases, but not across previous years.

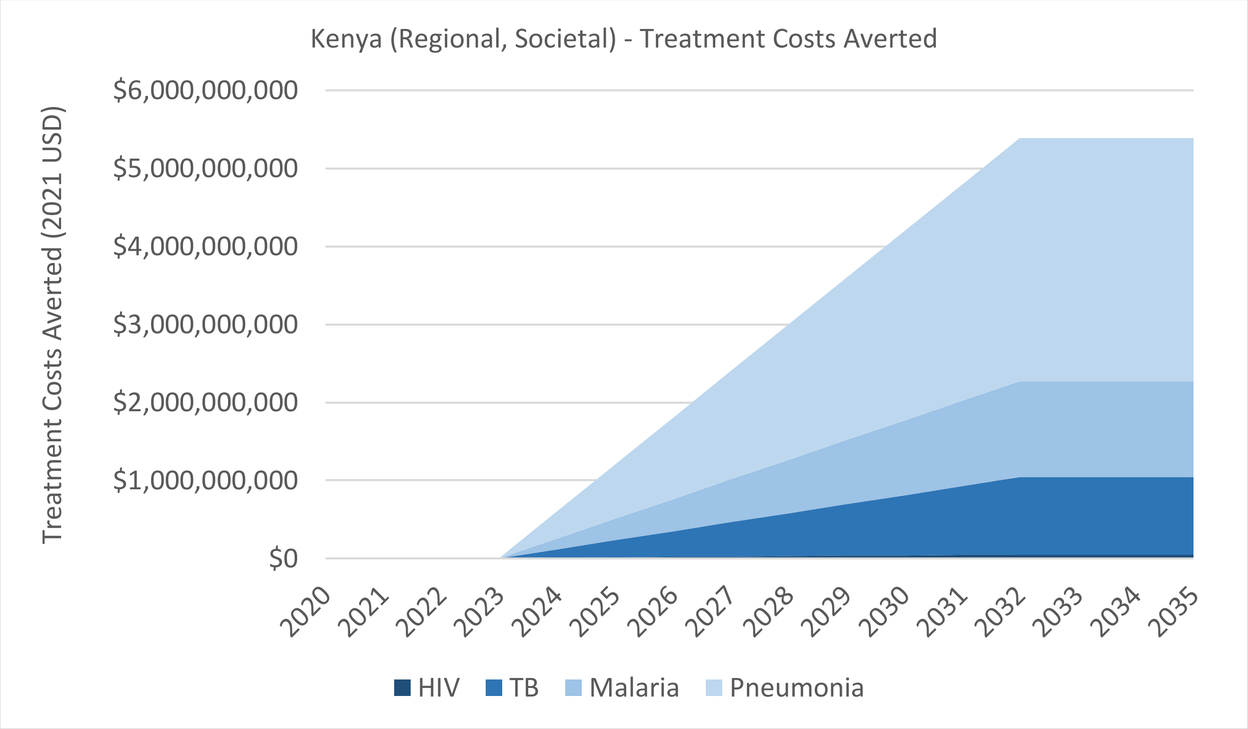

Figure A10. Treatment costs averted stratified by disease from the Kenya regional, societal perspective. The treatment costs averted in any given year are cumulative across diseases, but not across previous years.

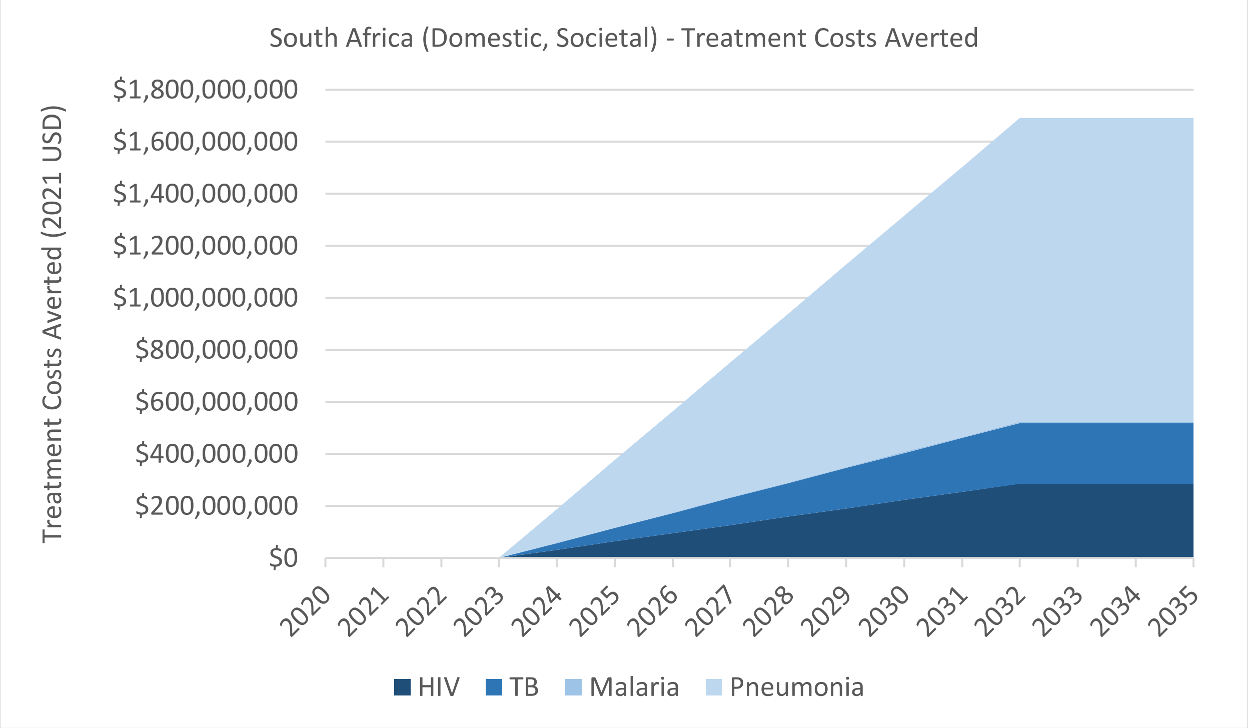

Figure A11. Treatment costs averted stratified by disease from the South Africa domestic, societal perspective. The treatment costs averted in any given year are cumulative across diseases, but not across previous years.

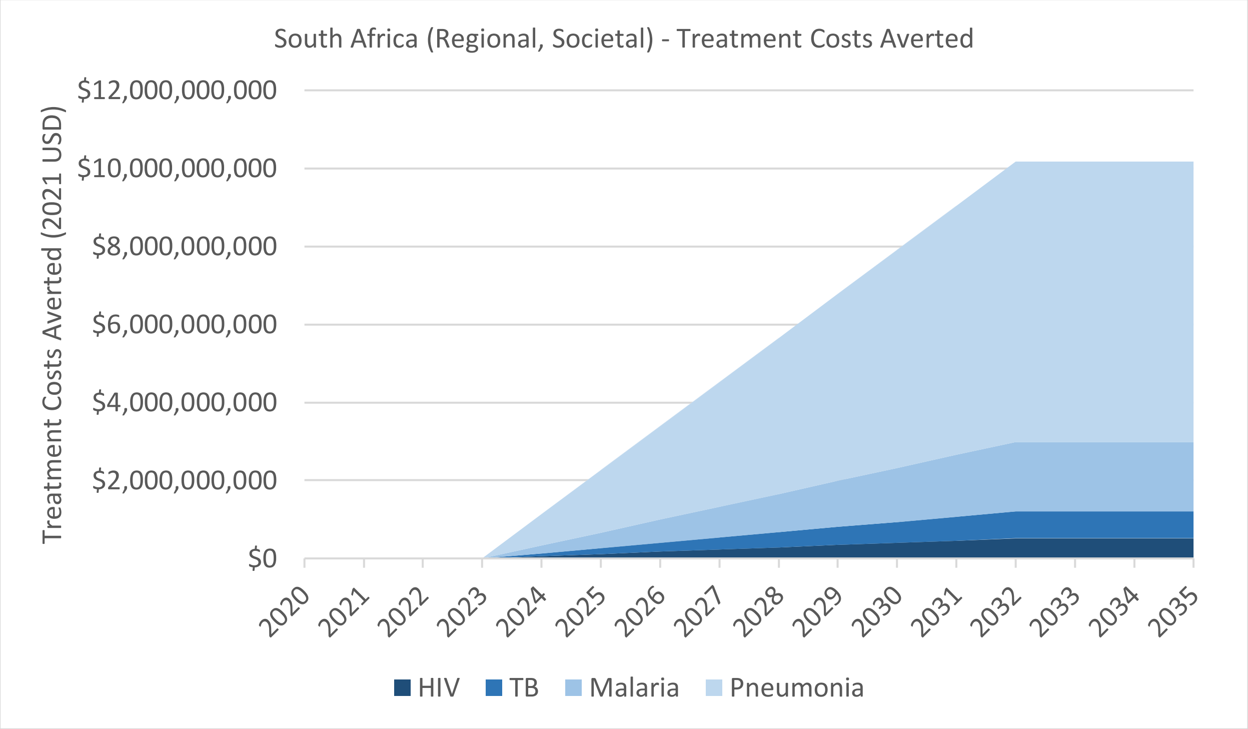

Figure A12. Treatment costs averted stratified by disease from the South Africa regional, societal perspective. The treatment costs averted in any given year are cumulative across diseases, but not across previous years.
